# Toward the identification of social signatures in ceramic production – An archaeological case study

**DOI:** 10.1371/journal.pone.0254766

**Published:** 2021-07-26

**Authors:** Ortal Harush, Leore Grosman

**Affiliations:** The Computational Archaeology Laboratory, The Hebrew University of Jerusalem, Mount Scopus, Jerusalem, Israel; University at Buffalo - The State University of New York, UNITED STATES

## Abstract

Ceramic analysis has been concerned with categorizing types according to vessel shape and size for describing a given material culture at a particular time. This analysis’ long tradition has enabled archaeologists to define cultural units across time. However, going into the analysis of sub-typological variations is rarely done, although their meanings bear significant consequences on the understanding of ties between individuals and social units. This study, aiming to assess whether it is possible to identify social signatures, focuses on a single archaeological ceramic type. For this propose, we selected a corpus of 235 storage jars from two distinct periods: storage jars from the Intermediate Bronze Age (the 25^th^ -20^th^ century BCE); and the Oval Storage Jar type (hereafter: OSJ) from the Iron Age II (the late 9^th^–early 6^th^ century BCE). The vessels selected were 3-D scanned to extract accurate geometric parameters and analyzed through an advanced shape analysis. The study results show that integrating computational and objective analysis methods, focusing on the “minute variation” within a single ceramic type, yields substantial insights regarding the relationship between variability and social units. In addition to the methodological guidelines and the suggested “work protocol” for further studies, the results shed light on the social organization of the Intermediate Bronze Age and the Iron Age II in Southern Levant.

## Introduction

Ceramic-sherds analysis has been concerned with categorizing sherds to vessel types based on form, function, and style as a means to define a given material culture at a specific time. Indeed, explaining similarity and change in ceramic vessels through time has been a long-standing goal among archaeologists [1–5]. Over the years, the general typologies that have been constructed ranged from describing complete assemblage in functional terms (jars, bowls, cooking pots, and others) to describing the geometric shapes of vessels (open/closed vessels) including an intra-observation of the vessels’ traits (carinated/straight profile, with neck/without, round/flat base, and others) [1, 5, 6]. These typologies are used to classify ceramic assemblages based on complete sets of vessels, with divisions made based on a general morphological average and depending on each researcher’s categorization system [7–12]. The traditional archaeological concept of dividing vessels into *types* and, at a higher resolution, their intra-division into *sub-types*, led to subjectivity and terminology related disagreements among scholars [13]. From a broader perspective, there seems to have been a lack of understanding concerning the causes underlying the subdivision of ceramic types. It has been suggested that understanding the causes for variations should be the object of archaeological inquiry, particularly explaining the reasons for variations within sub-types and their significance [14–16].

We have shown that integrating experimental and ethnographic datasets of sub-types within a defined ceramic type can be used to effectively detect collective [17] and individual morphological signatures [18]. Consequently, delving into the analysis of variations within a sub-type in archaeological assemblages could allow drawing conclusions beyond the general cultural definition. Such an analysis could highlight inter-group variability, enable the identification of social groups (e.g., community, village, workshop), and possibly allow the detection of intra-and inter-potter variability [17]. Thus, our goal in this high-resolution study is to apply our previously developed methodology [17, 18] on archaeological case studies in order to learn more on the connection between social dynamics and ceramic assemblages. We wish to test if morphometric analysis of ceramics can shed light on the causes underlying ceramic variations.

The latest advances in this domain suggest a novel approach based on 3-D scans that allow an automatic positioning procedure, which is objective, accurate and most importantly, consistent [19, 20]. This new method presents the considerable advantage of enabling the positioning of an unlimited number of ceramic sherds and, the production of accurate ceramic profiles, which can be analyzed through an automated classification procedure [17–19, 21–23]. The computational ceramic classification approach makes it possible to detect variations consistently and accurately by averaging formal ceramic types [19], and to point out the source of observed morphological variability [18].

Nevertheless, using morphometric features in archaeological ceramic assemblages as a proxy for the identification of social groups is not trivial, mainly because retrieved archaeological artifacts can rarely be tied to specific events but rather to prolonged time spans. Although traditional typological analysis can be used to detect general ceramic types, the premise of this study is to identify a more subtle aspect of long-term ceramic traditions—namely, cultural signatures. This term refers to the specific cultural fingerprint of groups that reflects their unique way of doing certain things [13]. The importance of this issue lies in the connection between morphological variation and cultural identity: the variations observed are directly related to the individual artisans’ cultural affiliation and technical traditions [24]. Specifically, individuals living in the same cultural, social, and physical environment tend, to a degree, to acquire a similar worldview, resulting in common behavioral traits that may be visible in material culture [14].

Several experiments have been conducted in the past in order to explain the meaning of variations within archaeological ceramic assemblages, and the relation of these variations to cultural context (e.g., [24]). According to these studies, the potters’ cultural affiliation affects how they produce their vessels [24], as a direct result of the selection of specific culturally situated motor skills [25]. This selection process creates a cultural marker, a signature, reflected in the final product.

As demonstrated in our previous publications [17], these aspects of morphological variability and trends in material culture can be explained in light of the Cultural Transmission Theory (henceforth CT) [14, 15, 26]. This theory is partially based on the typology and style-oriented approaches established by cultural historians to explain variation and similarities between artifacts [27–30]. Such approaches mostly aim to assess form, function, and style as means for periodization. CT, however, also focuses on other aspects of artifact variability, including behavior. The behavioral aspect of this theory relies on the notion that variability is highly affected by information exchange processes. The theory suggests that cultural features are reproduced with variations [31] due to a phenomenon called the copying error mechanism. This mechanism is defined as a small amount of error carried out in any copying event, resulting from physiological limitations in human perception and motor control, which prevents individuals from reproducing accurate model artifacts [15]. The main factor that affects the degree and type of copying error is the cultural setting of the artisan [24]. These unintentional variations, reflected in repeating morphometric traits, can be recognized through advanced morphological analysis, and serve as an anchor for analyzing minute variations [17, 18, 21, 22, 32]. Thus, when a significant morphometric distinction is observed in ceramic assemblages, it could suggest a meaningful regional and social trend.

The current research includes a precise analysis of variations in the morphology of ceramic vessels from two assemblages, focusing on one formal type (storage jars) to detect morphological regional and/or chronological trends. Furthermore, ethnographic research results [17] were used as reference data for interpreting morphometric variability in the archaeological ceramic assemblages.

We chose for this study two ceramic assemblages from the Southern Levant, differing in their established economic subsistence strategy and social structure: The Intermediate Bronze Age (henceforth IBA) and the Iron Age II (henceforth IR II).

Each period presents a unique opportunity to examine the variation in morphometric traits created through two different modes of production: The IBA production, domestic in nature, with no evidence of specialization [33–36]; and the IR II, manufactured and standardized [23, 32, 37, 38].

### The case studies

The first case study is composed of storage jars dating to the IBA, one of the most important periods for understanding the social and cultural processes that shaped Southern-Levant society throughout the Bronze Age. At the middle of the third millennium BCE, around 2500 BCE, there was a significant change in this region’s settlement pattern. Cities that thrived during the Early Bronze Age II–III (ca. 3100/3050–2450) (henceforth EB II–III) collapsed and were abandoned for a long time. The rebirth of urban society occurred only in the second millennium BCE (around 1900 BCE) (the Middle Bronze Age, henceforth MBA). In the transition from the EB III to the IBA, at the middle of the third millennium BCE, one of the significant changes was in the nature of the settlement, i.e., the population was now living in scattered villages [36, 39]. Consequently, most of this period’s available data comes from burial contexts or small settlements. Today, following a careful C14 study, it is widely accepted that the period began approximately in the 25^th^ century BCE and ended in the 20^th^ century BCE [40]. One of the great challenges in investigating this period is the difficulty in dividing it into sub-phases, resulting in an undifferentiated 500-years lapse. The pioneer researchers, Amiran and Dever [1, 41–43], provided the ceramic sub-division of this period based on regional and chronological criteria. According to these researchers, the early assemblages should be attributed to the northern region, arguably reflecting influences from Syria. Later assemblages then appeared in the Transjordan region, and from there they continued to gradually spread toward the south [43]. Although this scheme generates a great deal of confusion, experts of this period still use the dichotomous regional division set by Amiran [1, 41] and Dever [42, 43] to describe the IBA ceramic assemblages.

Amiran’s division is based on morphology and decoration in complete ceramic assemblages. She divided the ceramics of the period into four main families: ***A***, the Southern Family; ***B***, The Northern Family; ***C*** Black Wheel Made Ware (also known as “Megiddo Ware”); and ***D***, the Mountain Area, from Samaria to Jerusalem. She raised this regional division as a possible interpretation limited to the assemblages that were known in her time [1, 41]. In contrast, Dever divided the ceramics into six regional families [42, 43]: The North (***N***); the North–Central (***NC***); the Jordan Valley and Jericho (J); the South (***S***); the Central Hill (***CH***); and the Coast (***C***). It seems that his criteria were wider than those used by Amiran. In order to establish his division, he used not only ceramic assemblages, but also metal vessels and forms of burial. In general, Dever’s N family overlaps with Amiran’s B family and NC overlaps with Amiran’s C family. Dever’s J, CH, and S families are subdivisions of Amiran’s A family.

The second case study, dating to the Iron Age II, consists of the well-known *lmlk* and *Rosetta* jars. As early as 1953, Tuffnel [44] defined a unique type of jars that carry impressions, which has since been discussed in numerous publications as a key issue in the archeological and historical research of the Judaean Iron Age [45–49]. In 2006, Gittin named this group OSJ (Oval Storage Jar) [49], a comprehensive category for *lmlk* jars, *Rosetta* jars and similar jars without seal impression (including those Shai and Maeir called "Pre-*lmlk*" in 2003 [50]). In 2012, Sergi et al. [23] used the term OSJ as including both *lmlk* and *Rosetta* jars. The attribution of the two types (*lmlk* and *Rosetta*) to one general term (the OSJ type) and their association to the Kingdom of Judah’s administrative and economic system reflects their association to the same cultural group [23, 51, 52].

Although the current study uses a considerable part of the data from the article by Sergi et al. 2012 [23] and adopts the same methodology of shape analysis, the emphasis here is to reexamine the division between the two types and consider the socio-cultural implications of the variations observed.

Moreover, the assemblage analyzed here also includes some *lmlk* jars that bear no impressions, dated to the late 9^th^ century BCE (Horizon 1). Although such jars were previously referred to as pre- *lmlk* [49, 50], we propose to refer to these jars as part of the same ceramic tradition of the *lmlk* jars that lasted until the 7^th^ century BCE (Horizon 2–3) and the *Rosetta* type (Horizon 4) dated to the early 6^th^ century BCE [23].

The archaeological-data analysis was carried out on two different levels: An analysis of the ceramic profiles, and a classification of the jar assemblages with particular attention to their geographic spatial distribution. We aim to integrate these two factors and employ them as a tool, to not only assess their morphological variability but also identify morphological trends and traits from a regional perspective. Furthermore, the anthropological dataset [17] will serve as a comparative reference, and we will explore social and cultural aspects to interpret the variation within the archaeological ceramic assemblages.

## Material and method

### Body of data

#### The Intermediate Bronze Age storage-jar assemblage

**Fig 1:**
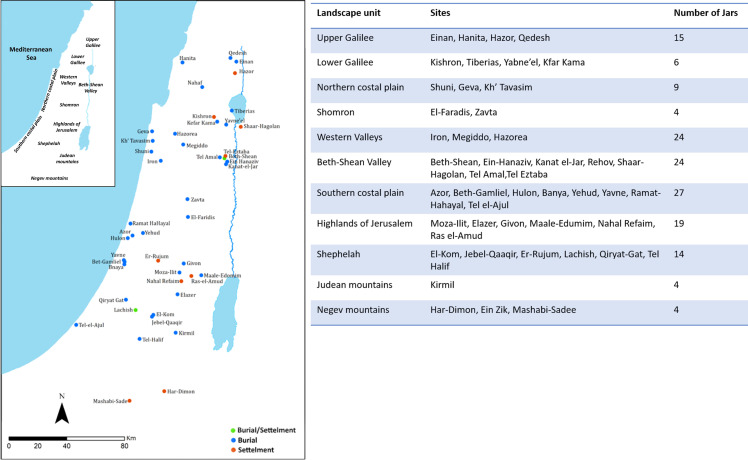
Distribution Map with ARCMAP of the IBA assemblages analyzed in this study (left), and the number of sites and jars per landscape unit (right). The jars’ contexts marked on the map: burial (blue dot); Settlement (red dot); and sites where jars originate from both contexts (green dot).

Our body of data includes 150 storage jars (S1 Table, DOI 10.17605/OSF.IO/ZMTSE) revealed in 48 sites from 11 landscape units (Fig 1) throughout the Southern Levant, discovered in burial contexts (N = 114), settlements (N = 25), or in sites where they were found in both contexts (N = 11). Due to the wide range of jar sizes, impeding a clear division between storage jars and jarlets, we selected only complete jars with over 10-liter storage capacity. The examined jars are described as either having a barrel-like or spherical/sac-like body [1, 36]. The majority have no handles (N = 80), followed in number by jars with shelf handles (N = 40), vertical handles (N = 13), and a single knob handle (N = 8). Jars with a flat base are the most frequent (N = 117), with a small group showing a round base (N = 33). All the jars are handmade, and bear applied elements (coils or slabs). Wheel marks were observed on the upper part of the jars, suggesting that the final styling, namely, the rim and neck, was performed on a slow wheel.

#### The Iron Age II lmlk and Rosetta assemblages (the OSJ group)

**Fig 2:**
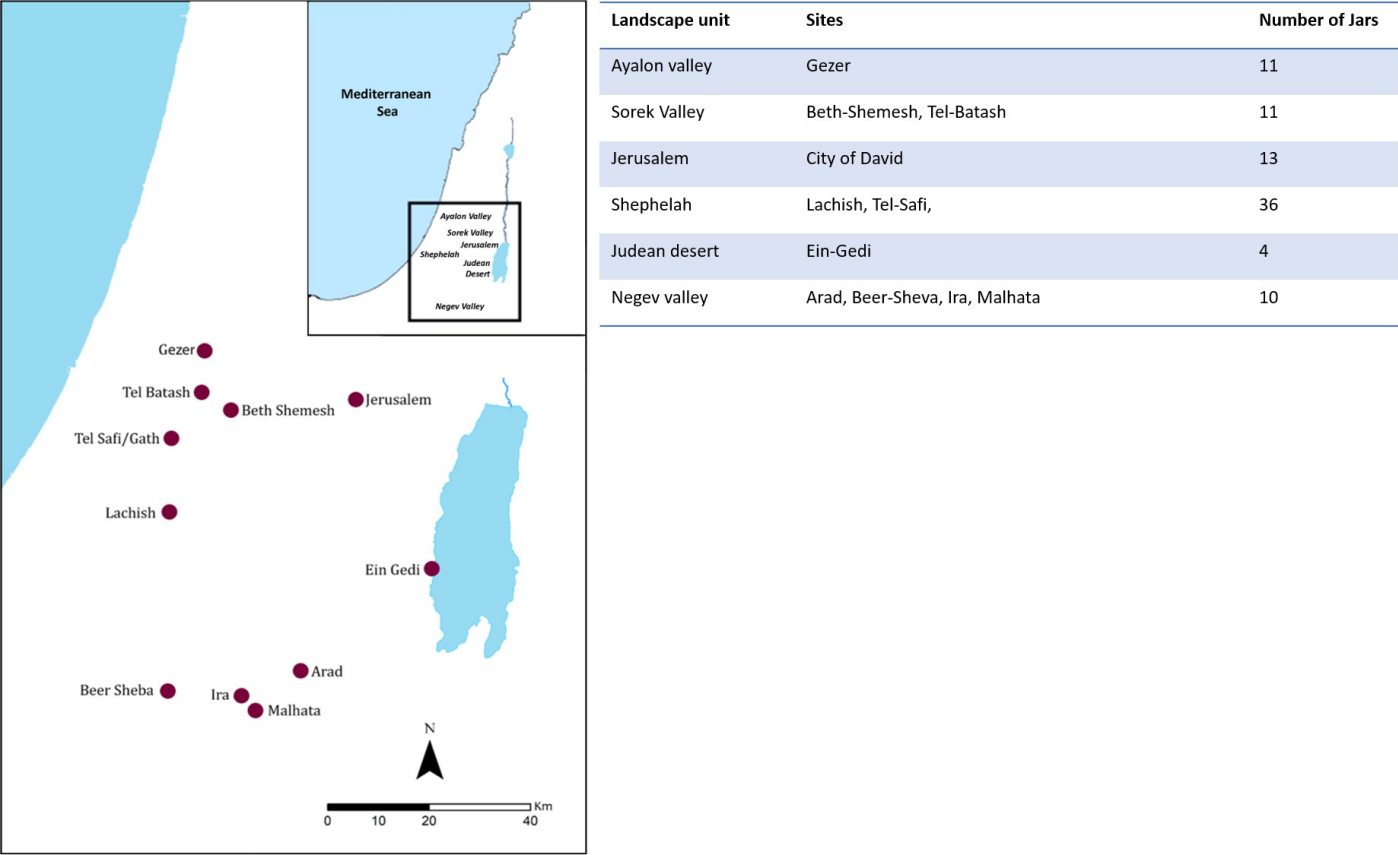
Distribution map with ARCMAP of the *lmlk* and the *Rosetta-*jar assemblages analyzed in this study (left), and the number of jars per landscape unit and site (right).

Our body of data includes 85 storage jars (S2 Table, DOI 10.17605/OSF.IO/ZMTSE) from 11 settlement sites (Fig 2) located in the Judean mountains and desert (N = 17), the Shephelah (N = 58), and the Negev (N = 10). The examined jars are complete or in an almost complete degree of preservation (comprising the shoulder and body but without a base). The Oval Storage Jars (OSJ) belong to two types: *lmlk* jars and *Rosetta* jars [23].

The database used here is partially borrowed from the study of Sergi et al. (2012) [23]. Additional jars were added to the study, mainly from an active excavation in Jerusalem, in order to increase the amount of *Rosetta* jars in the group.

Technological observation shows that both types are made in the wheel coiling technique. The body is built of coils, probably in two or three parts, and then shaped on a slow wheel.

### Three-dimensional (3-D) scanning

Three-dimensional models were used in this study to measure the morphological features of the archaeological ceramic assemblages. A total of 235 storage jars were scanned: 150 IBA vessels and 85 dated to the IR II.

Each vessel’s shape was captured using a structure-light scanner (Polymetric–PT-M Scanner), which produced high-resolution 3-D models [20]. The scanning was carried out at the Computational Archaeology Laboratory (The Hebrew University, Jerusalem, Israel), the Rockefeller Museum (Jerusalem, Israel) and The National Laboratory for Digital Documentation and Research (Israel Antiquities Authority, Jerusalem, Israel). The study also used 3-D models of IBA jars scanned with the DAVID-SLS2 scanner by Ron Lev, from the Kimmel Center at The Weizmann Institute.

### Data analysis

The morphological variability of the storage jars was analyzed across two data sets: The morphological classification of the vessel’s profiles [19], and the analysis of the rim’s radius and the rim- and neck-curve lengths [17, 18]. Furthermore, to verify and highlight the apparent relationship between regional trends and morphological types, we created density maps (Figs 1, 2, 6, 7 and 11) with *ARCMAP* showcasing these trends [53].

#### Morphological classification

The digitally based methods available in the *Pottery3-D* software (developed by Avshalom Karasik and Uzy Smilansky) were used to extract shape parameters for classifying the items based on their morphology [19, 54].

A cluster tree was generated based on the extracted parameters, providing a way to investigate grouping within the data. The distance measure in the cluster tree was based on three calculated mathematical functions for each profile: the radius (the size), the tangent (the general changes and trends of the shape), and the curvature (the minute changes throughout the profile). Together, these functions store the entire morphological information of the vessel’s profile [19, 54]. This makes it possible to process a large amount of data in a manner that resolves the subjectivity issue of visually formed typologies. After comparing all possible pairs of profiles in an assemblage, the outcome is a distance matrix that summarizes the similarities within the assemblage [19]. We applied two different weight values to fine-tune the classification procedure.

Since it is impossible to scan the inner profile of a complete jar, only the exterior profile of such jars was considered and measured.

#### Analysis of the rim’s radius and the rim- and neck-curve lengths

After positioning all the profiles in the *Pottery 3-D* software [19, 20], the measurements of the rim’s radius of each jar were obtained (extracted automatically from the *Pottery 3-D* program; see [20]).

We also applied the method to visualize the differences between ceramic profiles [18] and automatize the jars’ profile for two parts: Rim and Neck (abbreviated R and N). The R and N points were found by trimming the profile to the area of interest, followed by the smoothing of the profile curve and the automatic retrieval of the R and N points [17]. The segmentation of each portion allowed measuring different values throughout the profile. Indeed, after applying the automatic segmentation, we obtained measures for each part, which, together with the rim’s radius measures, enabled performing statistical significance tests.

## Results

### The Intermediate Bronze Age storage-jars assemblage

#### Profiles’ analysis

**Fig 3:**
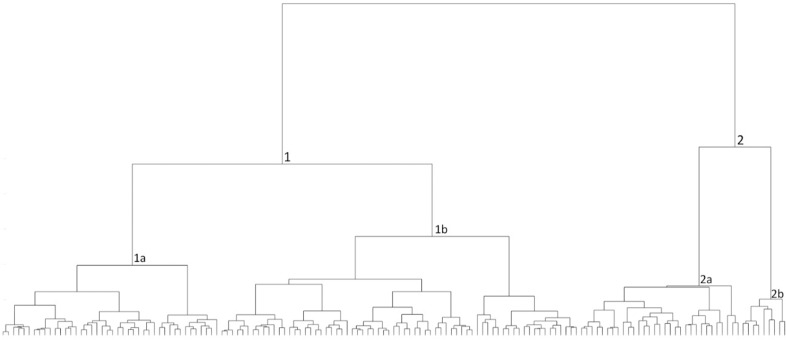
Cluster tree for the IBA 150 storage-jars. The lines at the base represent one item in the assemblage, and each branch represents a segregated morphological group (type variation) based on similarity.

A cluster tree was produced for the total IBA storage-jars assemblage (Fig 3) after applying the following weights: first round [20 70 10], second round [20 60 20]. The chosen weights (in order: radius, tangent, and curvature) emphasize the radius over curvature in the first step, and *vice versa* in the second iteration. The tangent function, found to be very significant during the morphological analysis, has a constant weight, between 60%–70%.

**Fig 4:**
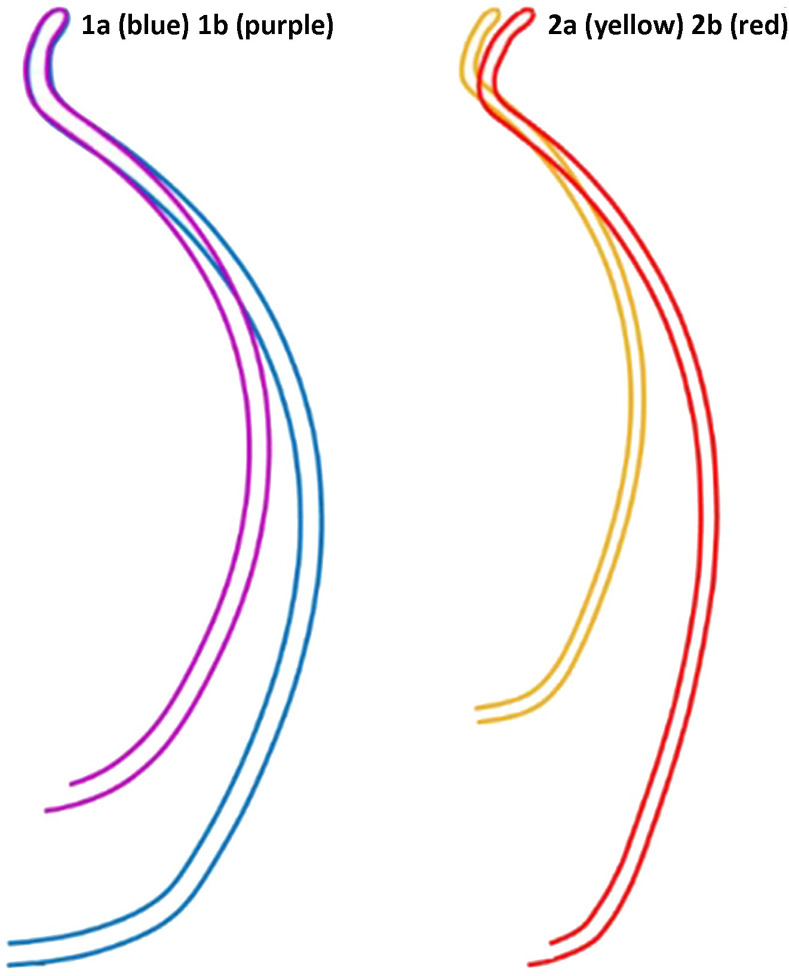
The average profiles of the two main branches (Types 1 and 2) and their subtypes (1a/b and 2a/b).

The automatic classification splits the assemblage into two distinct branches (types). The average profiles of the two main types (Fig 4) help visualize the clear division between them: Type1 (with two subtypes), a jar with a spherical body and a rounded and short neck; and Type 2 (with two subtypes), a jar with a barrel body and a flaring and high neck.

The differences between the two main branches (Types 1 and 2) and their subtypes stems from the variations in rim-radius values obtained from the *Pottery 3-D* software (Table 1) and from the rim- and neck-curve lengths obtained from the automation of the profile portions (Tables 2 and 3). In general, their values are significantly different (Mann-Whitney test, p<0.001); in particular, the rim-curve lengths show a difference between subtypes 1a and 1b, and between 1b and subtypes 2a and 2b. The rim’s radius and the neck-curve lengths show better significance, whereas Type 1 differs from Type 2. However, Subtypes 2a and 2b show significant differences in their values. The rim- and neck-curve lengths indicate a minute difference in each type’s profile. Type 1 has relatively shorter rim- and neck-curves, whereas those of Type 2 are longer.

**Table 1:** Significance test results for the rim’s radius among the subtypes.

	Type 1		Type 2	
	N = 111		N = 39	
	Subtype: 1a	Subtype: 1b	Subtype: 2a	Subtype: 2b
	N = 42	N = 69	N = 31	N = 8
	mean (mm): 58.97	mean (mm): 58.57	mean (mm): 64.25	mean (mm): 84.75
	STD: 5.83	STD: 5.64	STD: 6.15	STD: 12.65
Subtype: 1a	–	U = 1333.5	**U = 357**	**U = 0.5**
		p>0.99	**p<0.006**	**p<0.001**
Subtype: 1b		–	**U = 487**	**U = 5**
			**p<0.001**	**p<0.001**
Subtype: 2a			**–**	**U = 10**
				**p<0.0004**
Subtype: 2b				–

**Table 2:** Significance test results for the rim’s curve among the subtypes.

	Type 1		Type 2	
	N = 111		N = 39	
	Subtype: 1a	Subtype: 1b	Subtype: 2a	Subtype: 2b
	N = 42	N = 69	N = 31	N = 8
	mean (mm): 10.93	mean (mm): 9.35	mean (mm): 12.54	mean (mm): 15.29
	STD: 2.79	STD: 2.65	STD: 2.98	STD: 4.65
Subtype: 1a	–	**U = 905**	U = 449	U = 68
		**p<0.005**	p = 0.14	p = 0.05
Subtype: 1b		–	**U = 370**	**U = 49**
			**p<0.001**	**p<0.0009**
Subtype: 2a			–	U = 76
				p = 0.5
Subtype: 2b				–

**Table 3:** Significance test results for the neck’s curve among the subtypes.

	Type 1		Type 2	
	N = 111		N = 39	
	Subtype: 1a	Subtype: 1b	Subtype: 2a	Subtype: 2b
	N = 42	N = 69	N = 31	N = 8
	mean (mm): 35.03	mean (mm): 34.32	mean (mm): 42.07	mean (mm): 54.57
	STD: 10.46	STD: 11.02	STD: 7.68	STD: 9.5
Subtype: 1a	–	U = 1394	**U = 304**	**U = 28**
		p>0.99	**p<0.0006**	**p<0.001**
Subtype: 1b		–	**U = 445**	**U = 42**
			**p<0.001**	**p<0.0005**
Subtype: 2a			–	**U = 40**
				**p<0.02**
Subtype: 2b				–

#### The landscape unit scale

**Fig 5:**
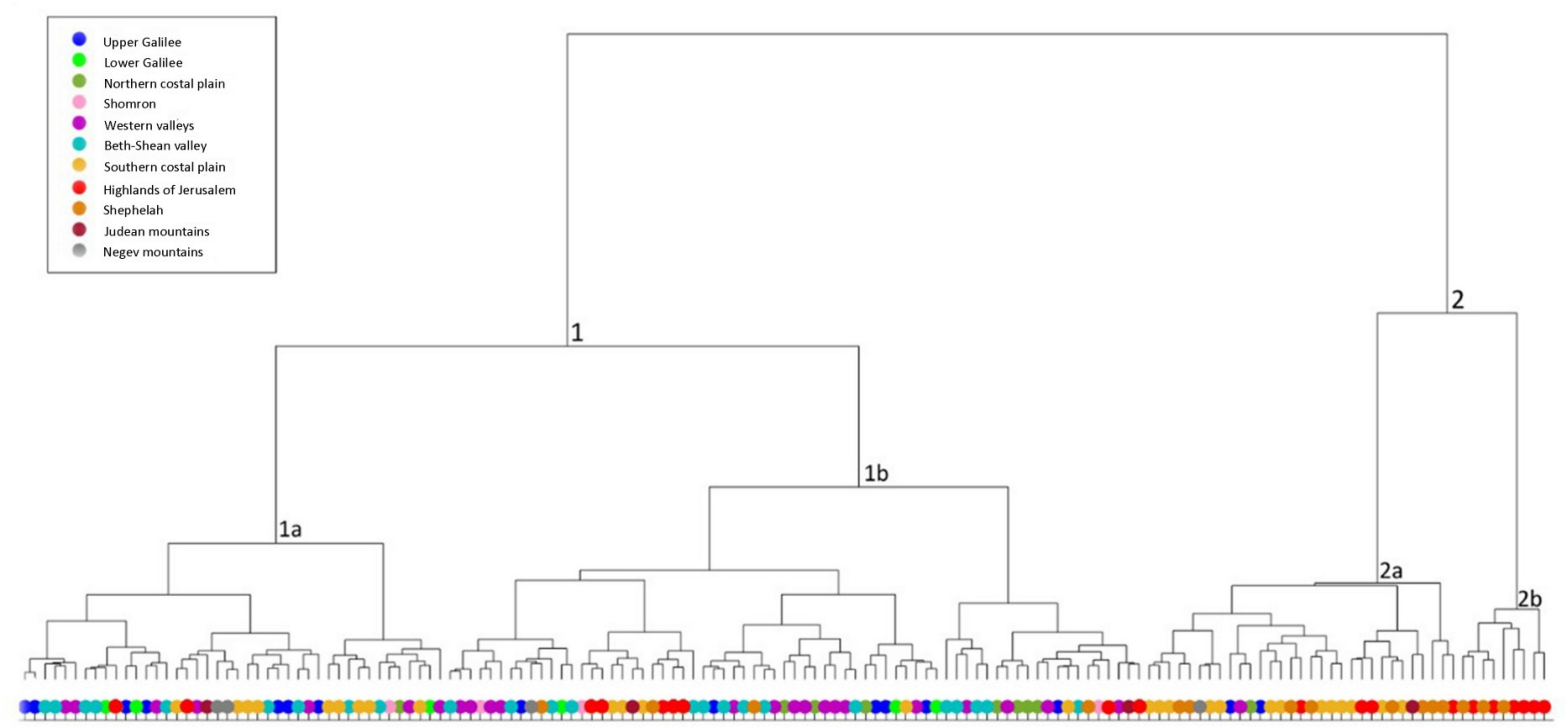
Cluster tree for the 150 IBA storage jars. The lines at the base represent one item in the assemblage, and each branch represents a segregated morphological group (type variation) based on similarity. The color-coded circles beneath the branches represent the group to which they belong (landscape units).

A cluster tree with landscape units was produced according to the weights mentioned above (Fig 5).

The automatic morphological classification divided the geographical units between the main branches and their subtypes. Type 1, marked in cold colors, comprises 76% of the North region, while Type 2, marked in warm colors, makes up 90% of the South region.

**Fig 6:**
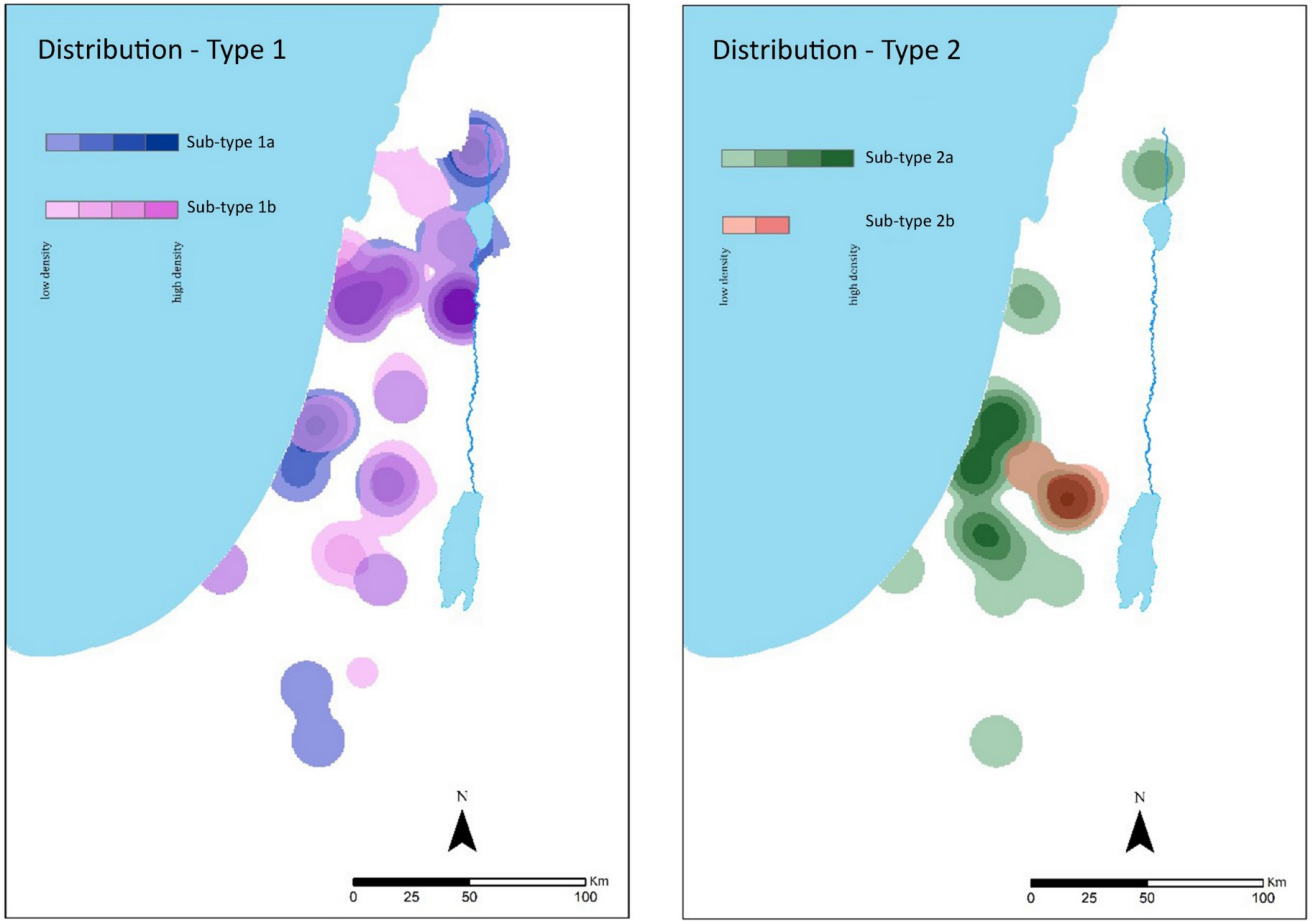
Density maps with ARCMAP showcasing the distribution of types 1 (left) and 2 (right) with their subtypes.

**Fig 7:**
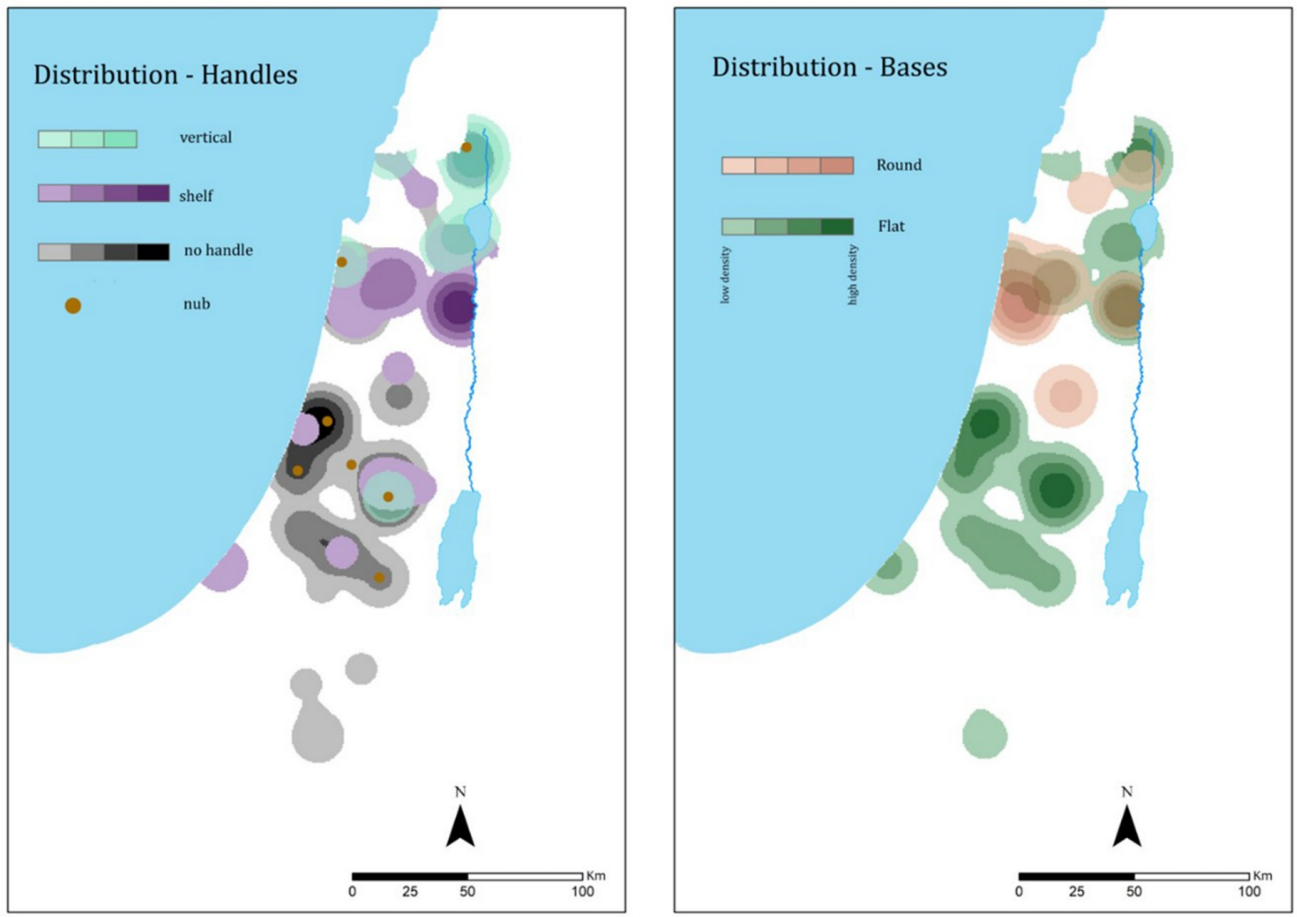
Density maps with *ARCMAP* showcasing the distribution of jars with handles (left) and different types of jar bases (right).

In general, Type 1 dominates the entire Southern Levant, concentrated mainly in the northern region (Fig 6: left). By splitting the type into its subtypes, it is possible to observe regional trends. Subtype 1a dominates the Beth-Shean valley, the upper Galilee and even the Southern coastal plain. Subtype 1b dominates the Beth-Shean valley and the western valleys, the lower and upper Galilee, the highlands of Jerusalem and the northern coastal plain. In this sense, Subtype 1b has a wider distribution than Type 1a. Type 2 differs from Type 1 not only in shape but also in distribution (Fig 6: right), as it is mainly concentrated in the Southern region. The number of jars of this type is not as high as in Type 1, but Type 2 is limited to the highlands of Jerusalem, the Shephelah and the southern coastal plain. A closer look at Type 2’s subtypes reveal that subtype 2b dominates mainly the highlands of Jerusalem (the majority coming from the sites of Nahal Refaim and Ras el-Amud), while 2a is found in the South and the Southern coastal plain. However, it is interesting to note that the jars from Moza-Illit, Givon, Elazar, and Maale-Adumim are clustered within the northern region Type 1 jars. Additional characteristics, such as the shape of the bases and the presence or lack of handles on the jars, were examined in relation to their geographical location (Fig 7).

Handles are generally present in both types (Fig 7: left). However, the dominance of handles in northern Type 1 stands out, together with the rounded shape of the jar’s base (Fig 7: right), a shape found only in the north region.

#### Insights on geographical variations

Amiran [1, 41] divided the ceramic IBA assemblages into four main regions: A, the Southern Family; B, The Northern Family; C Black Wheel Made Ware (also known as “Megiddo Ware”); and D, the Mountain Area, from Samaria to Jerusalem. Dever [43, 55], after Amiran, expanded the geographical division of the IBA into six regional families: The North (N); the North–Central (NC); the Jordan Valley and Jericho (J); the South (S); the Central Hill (CH); and the Coast (C). Both Amiran and Dever comparatively analyzed the IBA ceramic assemblages based on all ceramic typological types.

Their division somewhat overlaps (Dever’s N family = Amiran’s B family; Dever’s NC family = Amiran’s C family; and Dever’s J, CH, and S = family A). It is interesting to note that the Jerusalem area (characterized by Subtype 2b in the present study) was indeed defined as relating to a unique family (CH) in Dever’s division.

Despite the passage of time and many advances in research, i.e., the elongation of the period’s chronological sequence and new data from new excavations [40, 56–60], our automatic classification by shape supports Amiran’s general division and reveals itself also partly consistent with Dever’s regional subdivision. Thus, Dever’s regional division reinforces the results of the present study, with the two northern families he defined, N and NC, matching our Type 1 and its subtypes (1a and 1b). Also, Families C, CH, and S (Coastal Plain, Mountain, and Shephelah) are clustered in our Type 2, with Subtype 2a concentrated in the Shephelah and the Coastal Plain and 2b in the Mountain. It has not yet been determined whether these typological differences stem from different workshops, regional styles, or chronological considerations. However, a few interesting points should be considered:

Although the naked eye cannot always differentiate between Types 1 and 2 (Fig 3) the automatic classification of their profiles and related cluster tree (Fig 4) clearly distinguishes between them. Such a classification reflects the minute differences in the rim’s radius and the rim- and neck-curve lengths whose pattern varies across regions. These results may attest to two ceramic traditions, representative of two distinct social networks, highlighted by the morphometric grouping, perhaps resulting from different learning environments [24, 25, 61–64]. The morphometric features create cultural markers, as they are developed within localized learning niches, leading to differences in the jar’s profile morphometric features, which vary between the two main types.

We thus propose to consider these main types as representative of two social networks (types 1 and 2). The distribution of Type 1 is broader and spreads over many geographical units. However, the distribution of its subtypes demonstrates variation within the social network. These variations may arise from the involvement of many potters in the production process or distinct chronological phases that are notably unclear within the IBA.

In contrast, Type 2 is geographically limited. Here, the subtypes show a clear inter-regional division: Subtype 2a dominates mainly in the Shephelah and the Southern Coastal Plain, while Subtype 2b is found only in the Jerusalem highlands (particularly in Nahal Refaim and Ras el-Amud). It is noteworthy that burial sites within the Jerusalem highlands (Moza-Illit, Givon, Elazar, and Maale-Adumim) appear clustered in the northern ceramic tradition (Type 1), while settlement sites (Nahal Refaim and Ras el-Amud) belong distinctively to Subtype 2b. Interpreting this phenomenon is beyond the scope of this study, and we hope that future archaeological investigations will provide new data for understanding this unique pattern. The types’ distribution is also reflected in other attributes of the jars: The presence of handles and a rounded base is a northern Type 1 feature, while the southern Type 2 has a more ‘conservative’ and cohesive style.

### The Iron Age II Oval Storage Jar (OSJ): *Lmlk* and *Rosetta* jars

#### Profiles’ analysis

**Fig 8:**
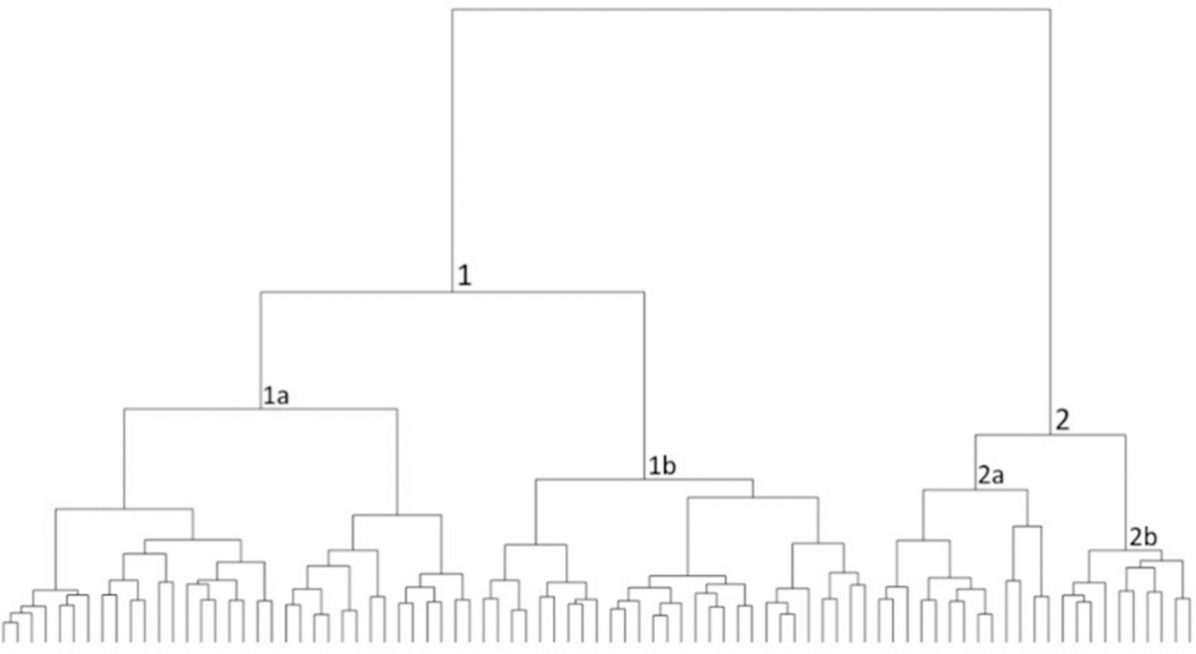
Cluster tree for 85 Iron Age II OSJ and *Rosetta* jars. The lines at the base represent one item in the assemblage, and each branch represents a segregated morphological group (type variation) based on similarity.

A cluster tree was produced for the complete OSJ assemblage (Fig 8) after applying the following weights: first round [10 70 20], second round [30 50 20]. The chosen weights (in order: the radius, the tangent, and the curvature) emphasize radius over the curvature in the first step, and vice versa in the second iteration. The tangent function, found to be very significant for the morphological analysis, has a constant weight, between 50%–70%.

**Fig 9:**
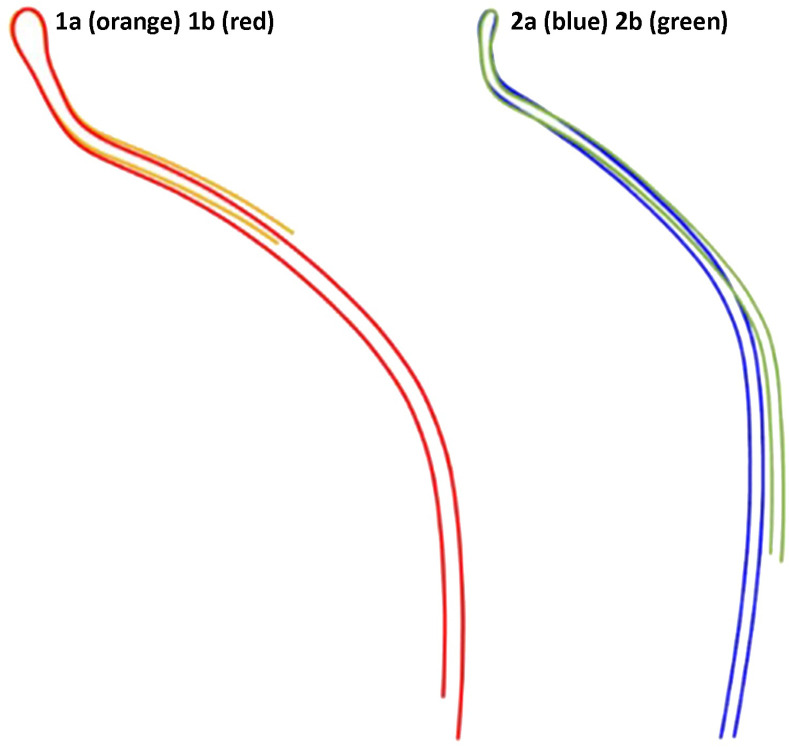
An average profile of the two main branches (Types 1 and 2) and their subtypes (1a, 1b and 2a, 2b).

The automatic classification splits the total assemblage into two distinct branches (types): Types 1 and 2, each with two subtypes. The average profiles of these two main types (Fig 9) help visualize the clear division between the two types: Type 1, representing the *lmlk* classical morphological jar type, and Type 2, which represents the *Rosetta* Jar type.

The differences between the two main branches (Types 1 and 2) and their subtypes stems from the variations in the rim-curve lengths obtained from the automation of the profile portions (Table 4). Although there is a clear significant difference in the rim-curve length, the rim radius values, and the neck-curve lengths show less substantial results.

**Table 4:** Significance test results for the rim radius, rim- and neck-curve lengths among the two main branches.

	Type 1: N = 62 Type 2: N = 23	Mean (mm)	STD	Mann-Whitney Test
Rim radius	Type 1	50.4	3.95	U = 676; d.f: 84; p = 0.0982
	Type 2	49.86	2.43	
**Rim curve**	Type 1	13.28	3.26	**U = 317; d.f = 84; p = 0.0002**
	Type 2	10.5	1.86	
Neck curve	Type 1	42.07	6.96	U = 550; d.f = 84; p = 0.1
	Type 2	39.66	8.55	

The results of the analysis of the rim- and neck-curve lengths values show that the rim curve in Types 1 and 2 is shorter than the neck curve. Particularly, Type 1 shows higher values, meaning the rim- and neck-curve lengths are greater than in Type 2.

#### The chronological-horizons scale

**Fig 10:**
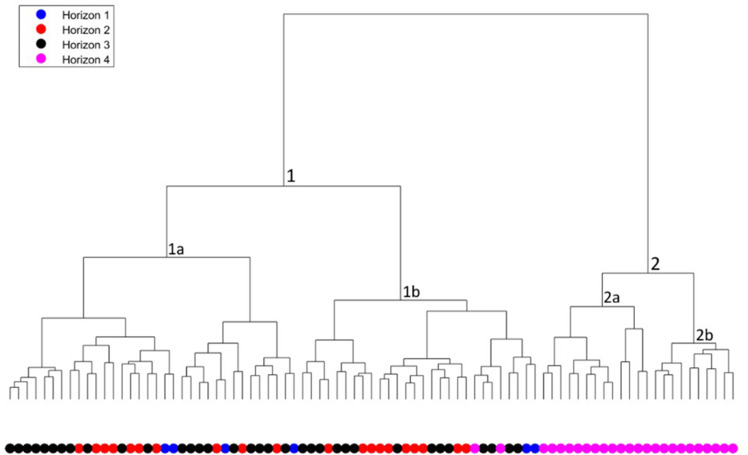
Cluster tree for the 85 Iron Age II OSJ and *Rosetta* jars with the chronological horizons. The lines at the base represent one item in the assemblage, and each branch represents a segregated morphological group (type variation) based on similarity. The color-coded circles beneath the branches represent the group/type’s chronological horizons to which they belong.

A cluster tree of chronological horizons was produced based on the weights mentioned above (Fig 10). The chronological contexts of the jars are the following: Horizon 1, dated to the late 9^th^ century BCE; Horizon 2, dated to the second third of the 8^th^ century BCE; Horizon 3, dated to the late 8^th^ century BCE; and Horizon 4, dated to the early 6^th^ century BCE [23].

The automatic morphological classification divided the chronological horizons, differentiating between main branches and their subtypes. Horizons 1–3, marked in red, black, and blue, belong to the Type 1 branch and are scattered throughout it, whereas Horizon 4, marked in pink, is present only in Type 2.

#### The landscape-unit scale

**Fig 11:**
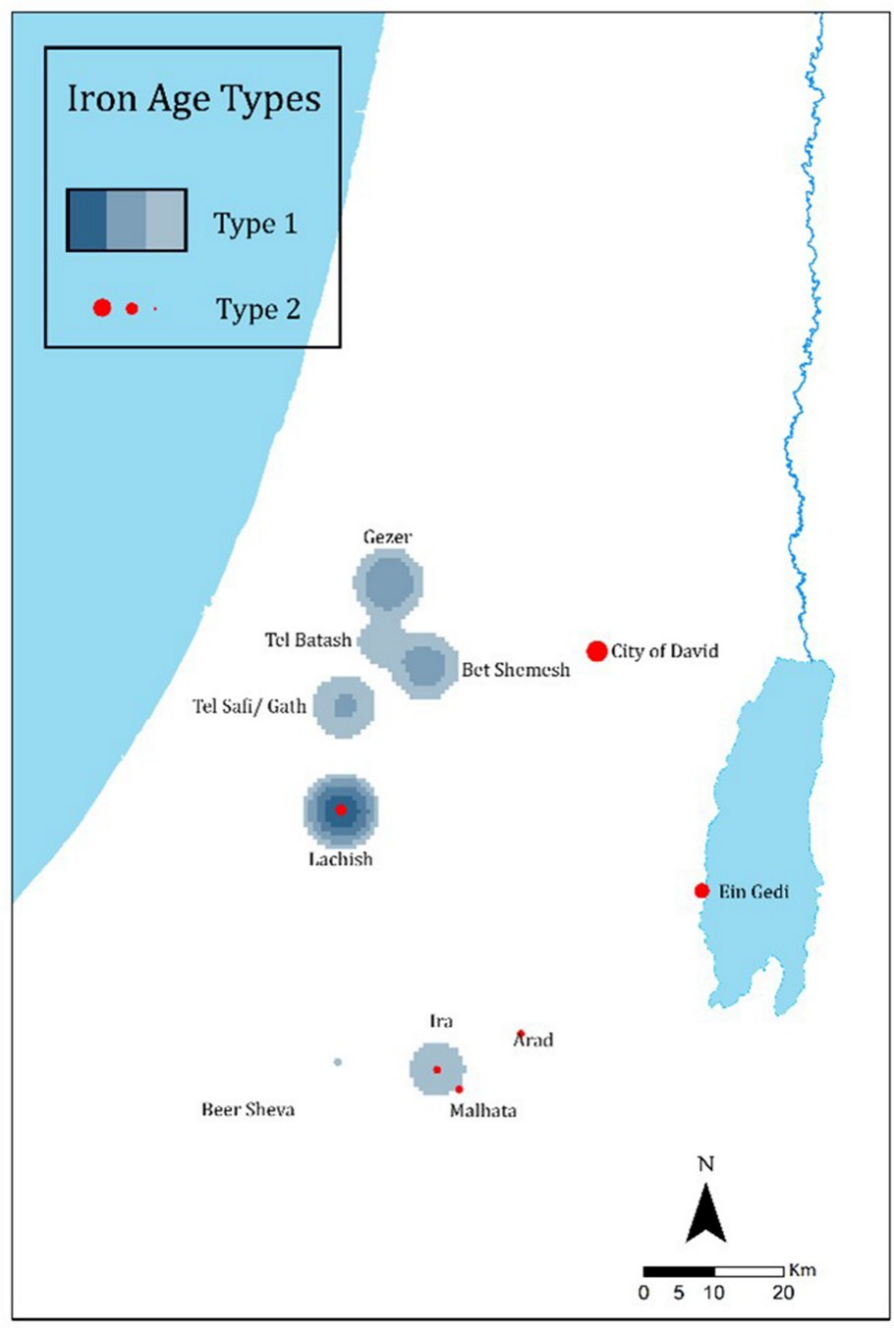
Density maps with *ARCMAP* showcasing the distribution of OSJ (Type 1) and *Rosetta* jars (Type 2).

The geographic distribution of the *lmlk* (Type 1) and the *Rosetta* jars (Type 2) shows distinct boundaries (Fig 11). Type 1, dominant in most of the Shephelah region up to the 7^th^ century BCE, was then replaced by Type 2, dominant mainly in the Jerusalem area.

#### Insights on the ceramic type variations

The *Rosetta* jars appearing in the late 7^th^ and early 6^th^ centuries BCE are known to have replaced the *lmlk* type [23, 51], being also considered a continuation of the *lmlk* tradition [23, 52]. This claim is based on the fact that the Instrumental Neutron Activation Analysis (INAA) and the petrographic results show that most of the *Rosetta* jars were made of the same clay used for the *lmlk* jars [23]. Indeed, these findings can hint at the same social unit, yet ethnographic studies show that different cultures can use the same clay recipe and producing technique and create the same type of vessel with different morphometric features, namely a different ‘style’ [19, 22]. Thus, the question arises as to why the *lmlk* jars’ form changed if both types belonged to the same tradition. The rim-curve lengths values are significantly different in the two types (Table 4), and their average profile (Fig 9) visually attests to these being two distinct types. Moreover, the correlation of these finds with the chronological horizons and geographical distribution shows a clear division between the two types. While the *lmlk*, defined here automatically as type 1, appears in the first three horizons in the Shephelah region, the *Rosetta*, type 2, appears only in the fourth horizon, mainly in Jerusalem. Continuity was suggested between these types [23, 51, 52] since both bear impressions and are produced with the same clay recipe. However, there is a c. 75-year gap between the *lmlk* jars’ disappearance in 7^th^ century BCE and the *Rosetta* jars’ appearance in the 6^th^ century BCE [51]. Also, based on the analysis above, each type has distinct morphometric features, suggesting perhaps gradual or abrupt change within this gap. If the change was indeed abrupt, it indicates the presence of a new social network. It is not clear whether a gradual change also manifests a transition in the social network.

## Discussion and conclusions

This study aimed to test whether it is possible to identify social signatures within the archaeological record. For this purpose, we examined two different datasets possessing established distinctive jar production systems: the IBA jars and the well-known *lmlk* and *Rosetta* jars from the IR II. In this last section of our text, we wish to discuss two main topics: the social signatures, and the significance of variability within the ceramic assemblage.

### The social signatures

In general, for all jars examined, the naked eye can rarely differentiate between variations, particularly within a single type. Yet the automatic classification and related cluster tree obtained in this study clearly distinguished between variation within a formal type that were supported through statistical tests. The classification reflects the minute differences in the vessels’ ceramic profile and emphasizes morphological trends that seem to correspond to regional and chronological variations. The automatic classification divided the IBA-jars data into two main branches (ceramic types), partly corresponding with Amiran’s [1, 41] and Dever’s [42, 43, 55] regional subdivision. Accordingly, we suggest ascribing the ceramic types (1 and 2) to two distinct social networks, i.e., cultural groups, each group with a distinct learning network affecting the morphometric groupings. Within the clustering results, we can distinguish differences in the network structure. The northern network is tightly embedded, with no inter-regional (sites) patterns. In contrast, the south network has at least two inter-regional signatures (the Shephelah and the Southern Coastal Plain, and the highlands of Jerusalem), which may indicate weak ties within the network [17, 65]. This configuration could be associated with a production network with several subnetworks resulting from potters not linked to each other by family ties.

According to latest publication on social signatures [17], which examined standardized production in two current-day endogamous communities manufacturing the same jar type using the same technique. The study was able to distinguish between the signatures of communities (social affiliation) and subgroups (villages and families). Our results in the present study show a similar behavior regarding network structure: an embedded community with one network vs. a diverse community with several (subnetworks).

The second case study presented, concerns the famous *lmlk* and *Rosetta* jar’s assemblages. Previous studies focused mostly on the impressions on their handles and linked the two types as one continuous tradition. In contrast, in our study, we found that each assemblage should be regarded as a distinct cultural ceramic tradition. The clear-cut separation between the *lmlk* and *Rosetta* types corresponds with the chronological and geographical data. While the *lmlk* type dominates the Shephelah for over 100 years, in the 7^th^ century BCE, its presence weakens, and a new type (the *Rosetta* jar) emerges, dominating most of the Jerusalem area. Historically, the point in time of this ceramic-tradition change (from the *lmlk* to the *Rosetta*) can be attributed to Sennacherib’s conquest in 701 BCE and subsequent weakening of the Shephelah [23, 32, 38, 39, 44, 46, 51, 65–69]. Before Sennacherib’s conquest, the *lmlk* jars were distributed and produced in this region. *Rosetta* jars emerge after Sennacherib’s conquest of the Shephelah and the deportation of the region’s population and are distributed mainly in Jerusalem and its surroundings until the Babylonian destruction of 586 BCE [51].

Despite the manifest gap between the appearance of the two types, and the uncertainty about whether it reflects an abrupt or gradual change from one ceramic type to the other, we propose to identify this shift from the *lmlk* type to the *Rosetta* type with a change in the social network. In other words, the two distinct types, highlighted by the morphometric grouping, might result from two social groups originating in different learning environments and creating cultural markers [19, 24, 25] reflected in the differentiated types.

### The significance of variability

Understanding the significance of variability within a ceramic assemblage is a major issue for characterizing ceramic production, enabling us to better approach the production organization, and, on a larger scale, the general social group organization, and the degree of craft specialization. The latter’s presence, an important component in the manufacturing process, points to standardization [70]. In general, standardized ceramic assemblages are associated with a high production rate. This correlation is the product of frequent practice improving skill and resulting in an increased standardization level of the products [12, 70–73]. Therefore, measuring the production rate, taking into account the standardization degree, enables evaluating the nature of the production and the social organization [73].

In present-day ceramic production [13, 17, 22, 65, 74] craft specialists with a high skill level can make 25 jars per day on average and work about 20 days per month, eight to ten months per year, corresponding to an annual production of 4000 to 6000 jars. Their regular rim-radius is reflected in their coefficient-variation statistical test (3%–6% range) [17]. All these factors point to a high-intensity standardized production.

**Fig 12:**
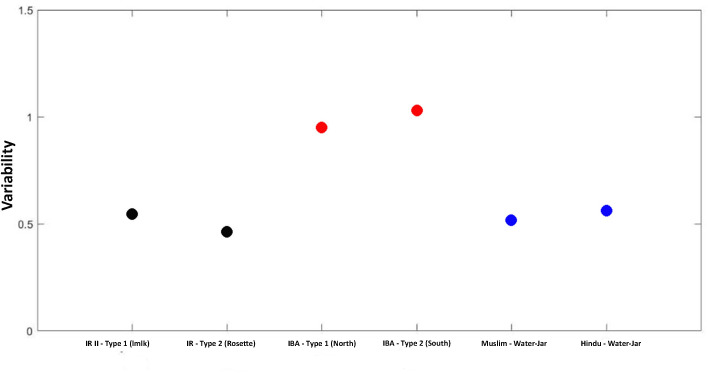
The variability degree of three datasets: The IR II (*lmlk* and *Rosetta* jars–black dots); the IBA jars (North and South–red dots); and the ethnographic reference data (Muslim and Hindu production–blue dots).

By comparing the variability degree (Fig 12) of the two datasets examined in this study with the ethnographic reference data [17], we can spot the variability degree within the archaeological record. The larger the values, the higher the degree of variability.

The IR II *lmlk* and *Rosetta*-jars assemblages show variability values similar to the ethnographic data, indicating a standardized production of the two ceramic types (Fig 12). Although each jar assemblage belongs to a different social network attested by the automatic classification above, they are all similar in skill level. In contrast, the IBA-jars assemblage (north and south) shows high variability values, indicating a less standardized and intensive production.

**Fig 13:**
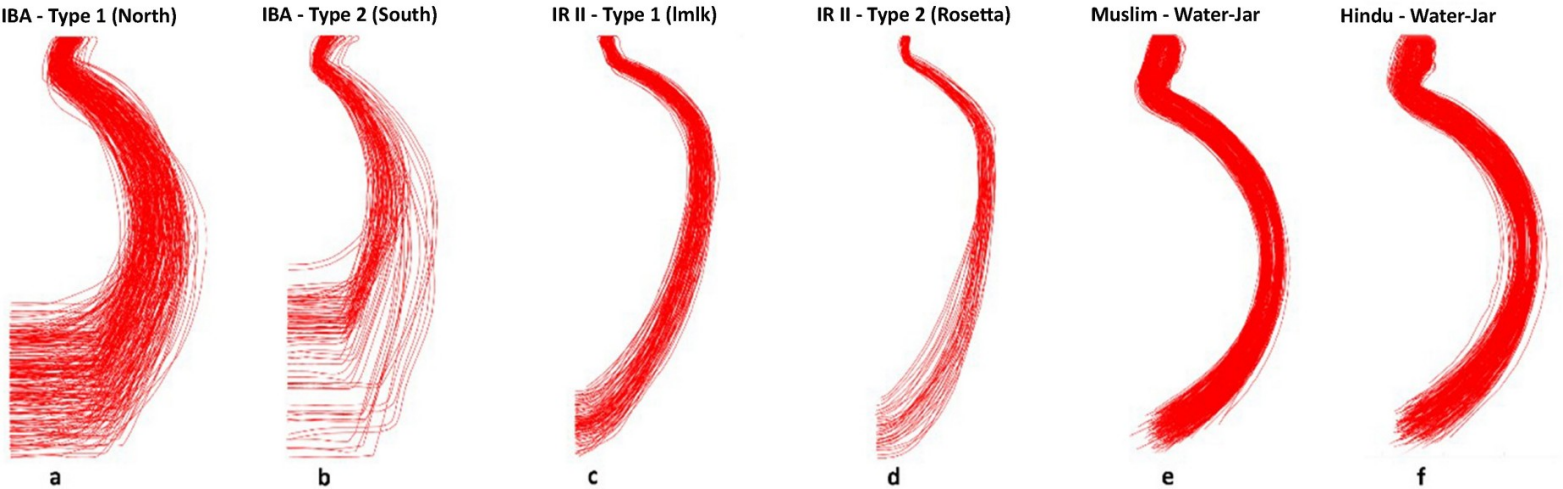
Overlapping of the jar’s profiles, from right to left: IBA jars, north type 1 (a); IBA jars, south type 2 (b); IR type 1 *lmlk* jars (c); IR type 2 *Rosetta* jars (d); Muslim water-jars (e); Hindu water-jars (f).

The overlapping of all the ceramic profiles from all groups (Fig 13) further verifies this production standard.

Within the IBA types (Fig 13A and 13B), the northern and southern types show much more diversity than the other groups (Fig 13C–13F), perhaps related to a different production organization and network structure of each social group. The *lmlk* and *Rosetta* types (Fig 13C and 13D) are regular and uniform, pointing to standardized production, a result strengthened by the side-by-side visual comparison with the ethnographic ceramic profiles (Fig 13E and 13F). The uniformity of the IR II jars has been widely discussed [23], and there is a general agreement on the period’s standardized nature of production. However, even within highly standardized assemblages, intra-variability occurs. The *lmlk* type shows uniformity along all three chronological horizons without distinct clusters per horizon, perhaps indicating a consistent and stable learning network through time. This network produces a standard jar type, and the variation reflects the number of potters involved in the production. The higher the number of potters involved, the more difficult it is to identify them or cluster them into meaningful groups [22]. In this case, the intra variability is reflected in the homogeneity of the *lmlk* profiles in all the chronological horizons, with no possibility to divide them into chronological phases.

As with the *lmlk* type, the *Rosetta* jars show regularity through the overlapping of the profiles. However, in contrast to the *lmlk*, they correspond only to the fourth horizon and a limited distribution area. Despite the low number of *Rosetta* jars examined in this study, these jars’ production seems to have been centralized and may have been carried out by a smaller number of potters than the *lmlk* assemblage.

In sum, although archaeologists are heavily invested in the typology of ceramics to construct diachronic sequences [75], achieving an overall chronological picture, little attention is given to ceramic variations within a sub-type and its significance. In the present study we were able to clearly differentiate between sub-typological ceramic variations, show regional (IBA) and chronological trends (IR II), and even point out the socio-cultural significance as to the differences that stood out.

Recently also, in other material cultural domains, there is growing interest in monitoring sub-social units. Metallurgy study showed that by analyzing change in the shape of metal production tools (crucibles, tuyères, and bellows), it was possible to highlight cultural and political changes in different magnitudes. Although attributed as a traditional and conservative craft, it was suggested that any change in the metalworking practice reflects sub-division between social networks [76]. This was also observed in animal remains analysis, which divided the diet to two animal species (cattle and figs) in the Southern Levant during the Bronze and Early Iron Age periods. Each type was distinct by place of origin (Aegean region vs. Egypt), and the route taken (sea vs. land) [77]. These results indicate the inter sub-division of animal remains, which reflect distinct social units.

Undoubtedly, future research should synthesis material remain such as—ceramics, metallurgy, fauna etc. Interpreting and investigating several technologies and materials are beyond the scope of this study, and we hope that future archaeological investigations will provide insights on this matter.

Thus, the importance of this study is in emphasizing minute changes in a single ceramic type. Indeed, differences between types and their division into groups are generally apparent in traditional archaeological studies, but the integration of computational and objective analysis methods emphasizes the differences within a single type and allows for a deeper understanding of the connection between variability and social units. Detecting craftsmen’s work requires looking for permanent and stable forms consistent throughout their appearance [18]. Thus, when observing a distinct and consistent pattern within an assemblage, we can be confident that it shows a real and meaningful social trend.

In this study, the analysis of the data benefited from a novel integration between quantitative and visualization methods and attention to trends and their geographic distribution. Integrating these methods succeeded in identifying trends within the morphological variability. The results show that social signatures can be highlighted given a careful analysis of minute variations within a ceramic type. Applied to a specific vessel type in a well-controlled archaeological dataset, our method, applicable to any ceramic assemblage, can help highlight various subgroups in an archaeological horizon where traditional methods reached an impasse.

## Supporting information

S1 Table Body of data: The Intermediate Bronze Age storage-jar assemblage. (PDF)

S2 Table Body of data: The Iron Age II lmlk and Rosetta assemblages (the OSJ group). (PDF)
