# Supplementary material for: Toward the identification of social signatures in ceramic production – An archaeological case study
Source: PLoS One. 2021 Jul 26;16(7):e0254766. doi: 10.1371/journal.pone.0254766 (PMC8312935; doi:10.1371/journal.pone.0254766)
Supplement: S1 Table — (PDF) [file pone.0254766.s001.pdf]

Site	Registration Number	Context	Landscape unit	Rim Radius	Rim Curve	Neck Curve	Base Radius	Exteranl vol	Completene	Morphologi	Handle type	Base type	Reference
Beth-Shean	32_174	Burial & Settelment	Beth-Shean Valley	62	8.822613	29.917026	57.72	27.92	complete	1a	shelf	round	Mazar, A. 2006. Tel Beth-Shean and the Fate of Mounds in the Intermediate Bronze Age. In Confronting the past: archaeological and historical essays on ancient Israel in honor of William G. Dever, 105–118. Eisenbrauns. Oren, ED. 1973. The northern cemetery of Beth Shan. Leiden: Brill.
Beth-Shean	I_9555	Burial & Settelment	Beth-Shean Valley	58	8.824889	43.416983	52.25	17.9	complete	1a	shelf	flat	Mazar, A. 2006. Tel Beth-Shean and the Fate of Mounds in the Intermediate Bronze Age. In Confronting the past: archaeological and historical essays on ancient Israel in honor of William G. Dever, 105–118. Eisenbrauns. Oren, ED. 1973. The northern cemetery of Beth Shan. Leiden: Brill.
Beth-Shean	I_9556	Burial & Settelment	Beth-Shean Valley	61	7.055966	38.513524	79.75	13.29	complete	1b	shelf	flat	Mazar, A. 2006. Tel Beth-Shean and the Fate of Mounds in the Intermediate Bronze Age. In Confronting the past: archaeological and historical essays on ancient Israel in honor of William G. Dever, 105–118. Eisenbrauns. Oren, ED. 1973. The northern cemetery of Beth Shan. Leiden: Brill.
Beth-Shean	I_9577	Burial & Settelment	Beth-Shean Valley	58	12.576972	32.553893	57.17	18.69	complete	1a	shelf	flat	Mazar, A. 2006. Tel Beth-Shean and the Fate of Mounds in the Intermediate Bronze Age. In Confronting the past: archaeological and historical essays on ancient Israel in honor of William G. Dever, 105–118. Eisenbrauns. Oren, ED. 1973. The northern cemetery of Beth Shan. Leiden: Brill.
Beth-Shean	V_1628	Burial & Settelment	Beth-Shean Valley	52	7.721059	28.211048	84.67	10.6	complete	1b	shelf	flat	Mazar, A. 2006. Tel Beth-Shean and the Fate of Mounds in the Intermediate Bronze Age. In Confronting the past: archaeological and historical essays on ancient Israel in honor of William G. Dever, 105–118. Eisenbrauns. Oren, ED. 1973. The northern cemetery of Beth Shan. Leiden: Brill.
Beth-Shean	V_1629	Burial & Settelment	Beth-Shean Valley	55	10.260486	68.332233	55.86	13.57	complete	1b	shelf	flat	Mazar, A. 2006. Tel Beth-Shean and the Fate of Mounds in the Intermediate Bronze Age. In Confronting the past: archaeological and historical essays on ancient Israel in honor of William G. Dever, 105–118. Eisenbrauns. Oren, ED. 1973. The northern cemetery of Beth Shan. Leiden: Brill.
Beth-Shean	183110-99	Burial & Settelment	Beth-Shean Valley	57	6.837379	31.739907	48.75	12.39	complete	1b	shelf	flat	Mazar, A. 2006. Tel Beth-Shean and the Fate of Mounds in the Intermediate Bronze Age. In Confronting the past: archaeological and historical essays on ancient Israel in honor of William G. Dever, 105–118. Eisenbrauns. Oren, ED. 1973. The northern cemetery of Beth Shan. Leiden: Brill.
Beth-Shean	580498-1	Burial & Settelment	Beth-Shean Valley	66	11.701037	31.817377	54.89	25.39	complete	1a	non	round	Mazar, A. 2006. Tel Beth-Shean and the Fate of Mounds in the Intermediate Bronze Age. In Confronting the past: archaeological and historical essays on ancient Israel in honor of William G. Dever, 105–118. Eisenbrauns. Oren, ED. 1973. The northern cemetery of Beth Shan. Leiden: Brill.
Beth-Shean	580522-1	Burial & Settelment	Beth-Shean Valley	62	7.660371	32.898124	55.27	20.73	complete	1a	non	round	Mazar, A. 2006. Tel Beth-Shean and the Fate of Mounds in the Intermediate Bronze Age. In Confronting the past: archaeological and historical essays on ancient Israel in honor of William G. Dever, 105–118. Eisenbrauns. Oren, ED. 1973. The northern cemetery of Beth Shan. Leiden: Brill.
Ein-Hanaziv	53-514	Burial	Beth-Shean Valley	61	8.779508	20.158644	57.05	22.05	complete	1a	shelf	flat	Porat, Y. 1972. Ein Hanaziv. Hadashot Arkheologiyot 44: 10.
Ein-Hanaziv	53-928	Burial	Beth-Shean Valley	57	7.164936	24.565247	55.24	12.84	complete	1b	shelf	flat	Porat, Y. 1972. Ein Hanaziv. Hadashot Arkheologiyot 44: 10.
Kanat-el-Jar	17-943	Burial	Beth-Shean Valley	58	7.181739	38.507665	52.95	10.08	complete	1b	shelf	flat	Horowitz, Z, & Atrash, W. 2016. Bet She'an, Tell Iztabba. Hadashot Arkheologiyot 128.
Kanat-el-Jar	17-944	Burial	Beth-Shean Valley	58	11.582308	37.307578	55.13	10.96	complete	1b	shelf	flat	Horowitz, Z, & Atrash, W. 2016. Bet She'an, Tell Iztabba. Hadashot Arkheologiyot 128.
Kanat-el-Jar	17-988	Burial	Beth-Shean Valley	64	8.37391	61.754894	90.86	18.06	complete	1a	shelf	flat	Horowitz, Z, & Atrash, W. 2016. Bet She'an, Tell Iztabba. Hadashot Arkheologiyot 128.
Kanat-el-Jar	73-1066	Burial	Beth-Shean Valley	65	8.648777	41.090406	90.74	15.74	complete	1b	shelf	flat	Horowitz, Z, & Atrash, W. 2016. Bet She'an, Tell Iztabba. Hadashot Arkheologiyot 128.
Kanat-el-Jar	73-1073	Burial	Beth-Shean Valley	52	10.474238	34.73883	53.38	14.16	complete	1b	shelf	flat	Horowitz, Z, & Atrash, W. 2016. Bet She'an, Tell Iztabba. Hadashot Arkheologiyot 128.
Kanat-el-Jar	73-1076	Burial	Beth-Shean Valley	54	8.701597	37.975288	54.47	13.54	complete	1b	shelf	round	Horowitz, Z, & Atrash, W. 2016. Bet She'an, Tell Iztabba. Hadashot Arkheologiyot 128.
Kanat-el-Jar	90-830	Burial	Beth-Shean Valley	55	7.198065	34.035133	53.06	10.66	complete	1b	shelf	flat	Horowitz, Z, & Atrash, W. 2016. Bet She'an, Tell Iztabba. Hadashot Arkheologiyot 128.
Kanat-el-Jar	90-963	Burial	Beth-Shean Valley	59	10.87449	63.180727	85.14	10.25	complete	1b	shelf	flat	Horowitz, Z, & Atrash, W. 2016. Bet She'an, Tell Iztabba. Hadashot Arkheologiyot 128.
Rehov	79-599	Burial	Beth-Shean Valley	53	7.072007	30.605989	51.51	13.38	complete	1b	shelf	flat	Yogev, O. 1985. A Middle Bronze Age Cemetery South of Tel Rehov. 'Atiqot 17 (ES) 17: 91–113.
Rehov	79-605	Burial	Beth-Shean Valley	59	8.966743	40.786843	100.85	22.19	complete	1a	shelf	flat	Yogev, O. 1985. A Middle Bronze Age Cemetery South of Tel Rehov. 'Atiqot 17 (ES) 17: 91–113.
Shaar-Hagolan	79-827	Settelment	Beth-Shean Valley	67	9.256512	31.575177	71.45	23.49	complete	1a	shelf	flat	Eisenberg, E. 2012. The Early Bronze Age IV site at Sha'ar Ha-Golan. Atiqot.
Tel-Amal	83-608	Burial	Beth-Shean Valley	64	11.657088	31.599365	54.94	13.61	complete	1b	shelf	flat	Feig, N. 1991. Burial Caves of the Early Bronze Age IV at Tel 'Amal. In Atiqot 20, 119–128. Israel Antiquities Authority.
Tel-Eztaba	17-177	Settelment	Beth-Shean Valley	64	7.617649	39.667697	87.03	17.34	complete	1b	shelf	flat	Horowitz, Z, & Atrash, W. 2016. Bet She'an, Tell Iztabba. Hadashot Arkheologiyot 128.
Elazer	K_8664	Burial	Highland of Jerusalem	62	13.267674	35.734532	47.32	12.81	complete	1b	non	flat	Gonen, R. 2001. Excavations At Efrata : A Burial Ground From the Intermediate and Middle Bronze Ages (IAA reports no. 12). Israel Antiquities Authority.
Givon	K_1648	Burial	Highland of Jerusalem	62	21.649359	29.607053	84.18	12.04	complete	1b	non	flat	Prichard, J. 1963. The Bronze Age Cemetery at Gibeon. Philadelphia.
Givon	K_4369	Burial	Highland of Jerusalem	60	11.575666	36.694411	55.48	12.72	complete	1b	non	flat	Prichard, J. 1963. The Bronze Age Cemetery at Gibeon. Philadelphia.
Givon	K_4370	Burial	Highland of Jerusalem	57	10.564164	35.748889	54.99	11.16	complete	1b	non	flat	Prichard, J. 1963. The Bronze Age Cemetery at Gibeon. Philadelphia.
Maale-Edomim	17864	Burial	Highland of Jerusalem	62	8.893269	43.487318	50.3	10.51	complete	1b	shelf	flat	Sion, O. 1995. Ma'alé Adummim. Hadashot Arkheologiyot 104: 126.
Moza-Ilit	72-153	Burial	Highland of Jerusalem	57	12.087041	37.840675	84.24	17.07	complete	1a	shelf	flat	Bahat, D. 1975. A Middle Bronze I Tomb-Cave at Motza. Eretz-Israel 12 12: 18–23.
Moza-Ilit	72-154	Burial	Highland of Jerusalem	47	7.065404	24.348472	50.12	19.67	complete	1a	shelf	flat	Bahat, D. 1975. A Middle Bronze I Tomb-Cave at Motza. Eretz-Israel 12 12: 18–23.
Moza-Ilit	99-710	Burial	Highland of Jerusalem	59	14.7386	31.659138	103.71	11.42	complete	1b	non	flat	Bahat, D. 1975. A Middle Bronze I Tomb-Cave at Motza. Eretz-Israel 12 12: 18–23.
Nahal Refaim	999_100_107_204	Settelment	Highland of Jerusalem	75	10.431805	42.803236	90.38	21.64	complete	2a	non	flat	Eisenberg, E. 1993. Naḥal Rephaim — A Bronze Age Village in Southwestern Jerusalem. Qadmoniot 3/4(103/104)): 82-95 (Hebrew).
Nahal Refaim	999_100_202	Settelment	Highland of Jerusalem	98	20.096268	60.006056	67.9	58.23	complete	2b	nub	flat	Eisenberg, E. 1993. Naḥal Rephaim — A Bronze Age Village in Southwestern Jerusalem. Qadmoniot 3/4(103/104)): 82-95 (Hebrew).
Nahal Refaim	999_100_203	Settelment	Highland of Jerusalem	75	20.968051	31.725605	54.02	32.11	complete	2a	non	flat	Eisenberg, E. 1993. Naḥal Rephaim — A Bronze Age Village in Southwestern Jerusalem. Qadmoniot 3/4(103/104)): 82-95 (Hebrew).
Nahal Refaim	999_100_3_1	Settelment	Highland of Jerusalem	75	13.611008	36.6187	56.98	20.64	complete	2a	vertical	flat	Eisenberg, E. 1993. Naḥal Rephaim — A Bronze Age Village in Southwestern Jerusalem. Qadmoniot 3/4(103/104)): 82-95 (Hebrew).
Nahal Refaim	999_115_151	Settelment	Highland of Jerusalem	55	8.316707	32.397737	96.46	12.78	complete	1b	non	flat	Eisenberg, E. 1993. Naḥal Rephaim — A Bronze Age Village in Southwestern Jerusalem. Qadmoniot 3/4(103/104)): 82-95 (Hebrew).
Nahal Refaim	999_505_40	Settelment	Highland of Jerusalem	86	16.877468	53.199195	57.82	46.94	complete	2b	non	flat	Eisenberg, E. 1993. Naḥal Rephaim — A Bronze Age Village in Southwestern Jerusalem. Qadmoniot 3/4(103/104)): 82-95 (Hebrew).
Nahal Refaim	999_506_87	Settelment	Highland of Jerusalem	82	12.020963	64.474152	53.24	50.66	complete	2b	non	flat	Eisenberg, E. 1993. Naḥal Rephaim — A Bronze Age Village in Southwestern Jerusalem. Qadmoniot 3/4(103/104)): 82-95 (Hebrew).
Nahal Refaim	999_725_775	Settelment	Highland of Jerusalem	61	9.013414	41.311196	103.47	23.34	complete	2a	non	flat	Eisenberg, E. 1993. Naḥal Rephaim — A Bronze Age Village in Southwestern Jerusalem. Qadmoniot 3/4(103/104)): 82-95 (Hebrew).
Nahal Refaim	999_862_295	Settelment	Highland of Jerusalem	84	11.607509	65.750461	57.45	48.46	complete	2b	non	flat	Eisenberg, E. 1993. Naḥal Rephaim — A Bronze Age Village in Southwestern Jerusalem. Qadmoniot 3/4(103/104)): 82-95 (Hebrew).
Ras-el-Amud	A5468-150.1	Settelment	Highland of Jerusalem	73	9.64603	40.909087	59.21	63.85	complete	2b	non	flat	Beeri, R, & Zilberbod, I. 2011. A Middle Bronze Age Settlement at Ras al-'Amud. *Qadmoniot* 142: 74-77 (Hebrew).

Ras-el-Amud	L527-5148-2	Settelment	Highland of Jerusalem	108	15.402064	47.205444	87.72	64.74	complete	2b	non	flat	Beeri, R, & Zilberbod, I. 2011. A Middle Bronze Age Settlement at Ras al-'Amud. *Qadmoniot* 142: 74-77 (Hebrew).
Kirmil	K130	Burial	Judean mountains	51	10.673969	39.721453	43.2	15.09	complete	1a	nub	flat	Dever, WG. 1975. A Middle Bronze I cemetery at Khirbet el-Kirmil. In Eretz-Israel 12, 18*-33*. Israel Exploration Society.
Kirmil	K131	Burial	Judean mountains	63	9.39948	31.111469	53.41	13.52	complete	1b	non	flat	Dever, WG. 1975. A Middle Bronze I cemetery at Khirbet el-Kirmil. In Eretz-Israel 12, 18*-33*. Israel Exploration Society.
Kirmil	K24	Burial	Judean mountains	63	6.692022	44.818139	87.14	10.79	complete	1b	non	flat	Dever, WG. 1975. A Middle Bronze I cemetery at Khirbet el-Kirmil. In Eretz-Israel 12, 18*-33*. Israel Exploration Society.
Kirmil	K25	Burial	Judean mountains	62	11.804404	53.780125	59.72	17.65	complete	2a	non	flat	Dever, WG. 1975. A Middle Bronze I cemetery at Khirbet el-Kirmil. In Eretz-Israel 12, 18*-33*. Israel Exploration Society.
Kefar-Kama	98-1533	Burial	Lower Galilee	56	7.480469	24.329314	80.1	16.45	complete	1b	shelf	flat	not published
Kefar-Kama	98-1534	Burial	Lower Galilee	51	8.930369	29.473764	50.31	12.21	complete	1b	vertical	flat	not published
Kishron	98-3484	Settelment	Lower Galilee	54	8.517727	33.639874	53.63	23.89	complete	1a	non	flat	Smithline, H. 2002. An Intermediate Bronze Age Site at Horbat Qishron. In Z. Gal (ed) Eretz Zafon, 20–47. Israel Antiquities Authority.
Tiberias	65-599	Burial	Lower Galilee	57	7.298872	22.917379	58.65	15.59	complete	1b	vertical	flat	Tzaferis, V. 1968. A Middle Bronze Age I Cemetery in Tiberias. Israel Exploration Journal 18(1): 15–19.
Tiberias	65-601	Burial	Lower Galilee	56	9.876207	26.569077	56.07	20.78	complete	1a	vertical	flat	Tzaferis, V. 1968. A Middle Bronze Age I Cemetery in Tiberias. Israel Exploration Journal 18(1): 15–19.
Yavne'el	80-377	Burial	Lower Galilee	66	15.73231	47.790871	58.93	24.13	complete	1a	shelf	flat	Leibowitz, H, & Porat, P. 1992. Early Bronze Age IV Tombs at Yavne'el. In 'Atiqot 21, 1–7. Israel Antiquities Authority.
Ein-Zik	85-777	Settelment	Negev mountains	63	12.405579	33.467471	51.21	11.31	complete	1a	non	flat	Cohen, R. 1999. Ancient Settlement in the Central Negev Y. Kuris (ed). Israel Antiquities Authority (Hebrew).
Ein-Zik	86-949	Settelment	Negev mountains	63	19.028024	27.661339	49.86	20.34	complete	2a	non	flat	Cohen, R. 1999. Ancient Settlement in the Central Negev Y. Kuris (ed). Israel Antiquities Authority (Hebrew).
Har Dimon	95-3926	Settelment	Negev mountains	81	11.393895	31.952292	76.25	16.96	complete	1b	non	flat	Cohen, R. 1999. Ancient Settlement in the Central Negev Y. Kuris (ed). Israel Antiquities Authority (Hebrew).
Mashabi-Sade	85-806	Settelment	Negev mountains	62	7.37677	35.132278	78.19	13.7	complete	1a	non	flat	Cohen, R. 1999. Ancient Settlement in the Central Negev Y. Kuris (ed). Israel Antiquities Authority (Hebrew).
Geva	70_7004	Burial	Northern coastal plain	52	7.280613	29.732244	49.91	11.43	complete	1b	shelf	round	Amiran, R. 1974. A Tomb-Group from Geva'-Carmel. In Atiqot: Hebrew Series, 1–12. (Hebrew)
Geva	70_7005	Burial	Northern coastal plain	51	8.760089	29.606009	44.46	14.56	complete	1b	non	round	Amiran, R. 1974. A Tomb-Group from Geva'-Carmel. In Atiqot: Hebrew Series, 1–12. (Hebrew)
Geva	70_7006	Burial	Northern coastal plain	51	8.25363	16.117827	57.24	17.17	complete	1b	vertical	flat	Amiran, R. 1974. A Tomb-Group from Geva'-Carmel. In Atiqot: Hebrew Series, 1–12. (Hebrew)
Geva	70_7007	Burial	Northern coastal plain	66	12.908028	32.949826	77.52	12.56	complete	1b	nub	flat	Amiran, R. 1974. A Tomb-Group from Geva'-Carmel. In Atiqot: Hebrew Series, 1–12. (Hebrew)
Kh-Tavasim	00-662	Burial	Northern coastal plain	63	7.155688	28.732793	50.9	9.98	complete	1b	non	round	Hess, O. 1980. A Middle Bronze Age I burial cave at Fureidis. In Atiqot 14, 34–36. Israel Antiquities Authority.
Kh-Tavasim	00-663	Burial	Northern coastal plain	66	8.463032	31.649666	53.25	13.05	complete	1b	non	round	Hess, O. 1980. A Middle Bronze Age I burial cave at Fureidis. In Atiqot 14, 34–36. Israel Antiquities Authority.
Shuni	T-32-30613	Burial	Northern coastal plain	64	13.760675	30.757945	50.69	21.5	complete	1a	non	round	Peilstöcker, M, & Sklar-parnes, DA. 2005. ' Enot Shuni. Hadashot Arkheologiyot 117(December 2001).
Shuni	T-85-7312	Burial	Northern coastal plain	61	7.682686	27.596248	50.9	11.01	complete	1b	non	round	Peilstöcker, M, & Sklar-parnes, DA. 2005. ' Enot Shuni. Hadashot Arkheologiyot 117(December 2001).
Shuni	T-90-7937	Burial	Northern coastal plain	75	10.29073	36.742091	60.95	27.5	complete	2a	non	round	Peilstöcker, M, & Sklar-parnes, DA. 2005. ' Enot Shuni. Hadashot Arkheologiyot 117(December 2001).
El-Kom	EQ219	Burial	Shfela	58	11.242745	36.56324	45.87	14.3	complete	1b	non	flat	Dever, WG. 2014. Excavations at the Early Bronze IV Sites of Jebel Qa'aqir and Be'er Resisim. Winona Lake.
Er-Rujum	2172	Settelment	Shfela	66	10.227621	34.268089	10.37	37.66	complete	2a	non	flat	Milevski, I et al. 2012. Er-Rujum (Sha'alabim East): An Intermediate Bronze Age (EB IV) Site in the Ayyalon Valley. In Atiqot 69, 75–140. Israel Antiquities Athority.
Er-Rujum	2203	Settelment	Shfela	72	23.39911	44.515732	118.82	47.16	complete	2b	nub	flat	Milevski, I et al. 2012. Er-Rujum (Sha'alabim East): An Intermediate Bronze Age (EB IV) Site in the Ayyalon Valley. In Atiqot 69, 75–140. Israel Antiquities Athority.
Er-Rujum	2215-2	Settelment	Shfela	75	13.323789	60.542815	100.06	39.84	complete	2b	non	flat	Milevski, I et al. 2012. Er-Rujum (Sha'alabim East): An Intermediate Bronze Age (EB IV) Site in the Ayyalon Valley. In Atiqot 69, 75–140. Israel Antiquities Athority.
Jebel-Qaaqir	1074	Burial	Shfela	53	12.789516	25.196508	46.77	13.33	complete	1b	shelf	flat	Dever, WG. 2014. Excavations at the Early Bronze IV Sites of Jebel Qa'aqir and Be'er Resisim. Winona Lake.
Jebel-Qaaqir	Q170	Burial	Shfela	51	7.47927	27.841216	49.57	11.24	complete	1b	non	flat	Dever, WG. 2014. Excavations at the Early Bronze IV Sites of Jebel Qa'aqir and Be'er Resisim. Winona Lake.
Jebel-Qaaqir	Q193	Burial	Shfela	66	12.02701	48.229623	84.7	22.47	complete	2a	non	flat	Dever, WG. 2014. Excavations at the Early Bronze IV Sites of Jebel Qa'aqir and Be'er Resisim. Winona Lake.
Jebel-Qaaqir	Q91	Burial	Shfela	50	7.159693	36.355917	89.58	10.68	complete	1b	non	flat	Dever, WG. 2014. Excavations at the Early Bronze IV Sites of Jebel Qa'aqir and Be'er Resisim. Winona Lake.
Lachish	34_2911	Burial & Settelment	Shfela	65	8.540449	49.181261	89.97	17.19	complete	2a	non	flat	Tufnell, O. 1958. Lachish IV: The Bronze Age. Oxford University Press.
Lachish	34_2916	Burial & Settelment	Shfela	68	11.475723	50.925772	94.02	21.85	complete	2a	non	flat	Tufnell, O. 1958. Lachish IV: The Bronze Age. Oxford University Press.
Qiryat-Gat	1000	Burial	Shfela	69	15.795307	42.850035	97.09	18.27	complete	2a	non	flat	Abadi-reiss, Y. 2019. Qiryat Gat. Hadashot Arkheologiyot 131: 15.
Qiryat-Gat	1005	Burial	Shfela	62	10.121966	45.536895	49.64	23.88	complete	2a	non	flat	Abadi-reiss, Y. 2019. Qiryat Gat. Hadashot Arkheologiyot 131: 15.
Qiryat-Gat	1007	Burial	Shfela	62	14.953479	49.644119	100.3	18.62	complete	2a	non	flat	Abadi-reiss, Y. 2019. Qiryat Gat. Hadashot Arkheologiyot 131: 15.
Tel-Halif	72-15	Burial	Shfela	61	10.816859	45.291326	45.96	21.11	complete	2a	non	flat	Alon, D. 1973. Tel Halif. Hadashot Arkheologiyot 47: 19-20 (Hebrew).
El-Faridis	74-1563-1	Burial	Shomron	60	16.760873	31.282203	48.5	12.55	complete	1b	non	round	Horowitz, Z, & Masrowa, M. 1999a. Baqa al-Gharbiya. Hadashot Arkheologiyot 110: 115.
El-Faridis	74-1566	Burial	Shomron	65	15.026126	34.398083	50.24	21.27	complete	1a	non	round	Horowitz, Z, & Masrowa, M. 1999a. Baqa al-Gharbiya. Hadashot Arkheologiyot 110: 115.
El-Faridis	74-1573	Burial	Shomron	55	7.127696	32.040031	52.46	11.38	complete	1b	non	round	Horowitz, Z, & Masrowa, M. 1999a. Baqa al-Gharbiya. Hadashot Arkheologiyot 110: 115.
Zavta	K_17864	Burial	Shomron	60	10.33745	60.871077	97.07	12.04	complete	1b	shelf	flat	not published
Azor	56-25	Burial	Southern Coastal plain	55	16.553269	31.647176	89.09	16.97	complete	1a	non	flat	Yannai, E. 2007. Burial Ground from the intermediate Bronze Age at Azor. Atiqot 55: 1-28 (Hebrew).
Azor	62-76	Burial	Southern Coastal plain	62	12.363431	41.350369	59.64	22.34	complete	2a	non	flat	Yannai, E. 2007. Burial Ground from the intermediate Bronze Age at Azor. Atiqot 55: 1-28 (Hebrew).
Azor	64-579	Burial	Southern Coastal plain	49	10.600264	31.346419	48.35	16.63	complete	1a	non	flat	Yannai, E. 2007. Burial Ground from the intermediate Bronze Age at Azor. Atiqot 55: 1-28 (Hebrew).
Azor	71-32	Burial	Southern Coastal plain	54	12.701102	30.839987	49.45	15.45	complete	1a	non	flat	Yannai, E. 2007. Burial Ground from the intermediate Bronze Age at Azor. Atiqot 55: 1-28 (Hebrew).
Bet-Gamliel	61-521	Burial	Southern Coastal plain	46	11.574141	32.618837	40.49	19.83	complete	1a	non	flat	not published
Bet-Gamliel	61-522	Burial	Southern Coastal plain	50	11.070799	43.549321	47.82	21.2	complete	2a	non	flat	not published
Bnaya	62-112	Burial	Southern Coastal plain	69	11.137194	44.645711	93.15	22.54	complete	2a	nub	flat	not published
Bnaya	62-99	Burial	Southern Coastal plain	66	16.5549	40.916424	56.57	17.69	complete	2a	non	flat	not published
Bnaya	70-113	Burial	Southern Coastal plain	53	10.413557	32.174598	50.78	25.03	complete	1a	non	flat	not published
Hulon	64-572	Burial	Southern Coastal plain	53	9.920501	37.499755	88.58	11.29	complete	1b	non	flat	Negbi, O. 1975. Holon (Industrial Zone). Hadashot Arkheologiyot 56: 29-30 (Hebrew).
Hulon	64-618	Burial	Southern Coastal plain	54	14.497703	30.102273	59.13	22.91	complete	1a	shelf	flat	Negbi, O. 1975. Holon (Industrial Zone). Hadashot Arkheologiyot 56: 29-30 (Hebrew).
Hulon	64-622	Burial	Southern Coastal plain	55	13.0277	35.754322	58.33	20.18	complete	2a	non	flat	Negbi, O. 1975. Holon (Industrial Zone). Hadashot Arkheologiyot 56: 29-30 (Hebrew).
Ramat-Hahayal	17-1049	Burial	Southern Coastal plain	58	11.810839	32.611954	51.95	24.2	complete	2a	non	flat	Yankelevitch, S. 2004. A Salvage Excavation at Ramat-HaHayal, Tel Aviv. In Contract Archaeology Reports I, 27–38. Haifa: Rekanaty Institute for Maritime Studies
Tel el_Ajul	32_2223	Burial	Southern Coastal plain	58	10.382531	18.477664	58.42	16.78	complete	1b	shelf	flat	Kenyon, KM. 1956. Tombs of the Intermediate Early Bronze- Middle Bronze at Tell el-Ajjul. In Annual of the Department of Antiquities of Jordan 3, 41–55.
Tel el_Ajul	35_3795	Burial	Southern Coastal plain	56	10.759368	48.176453	54.61	26.44	complete	2a	shelf	flat	Kenyon, KM. 1956. Tombs of the Intermediate Early Bronze- Middle Bronze at Tell el-Ajjul. In Annual of the Department of Antiquities of Jordan 3, 41–55.
Tel el_Ajul	I_10107	Burial	Southern Coastal plain	74	9.08939	33.157139	87.9	12.12	complete	1b	non	flat	Kenyon, KM. 1956. Tombs of the Intermediate Early Bronze- Middle Bronze at Tell el-Ajjul. In Annual of the Department of Antiquities of Jordan 3, 41–55.
Tel el_Ajul	I_10117	Burial	Southern Coastal plain	57	12.474304	42.355439	92.41	20.11	complete	1a	non	flat	Kenyon, KM. 1956. Tombs of the Intermediate Early Bronze- Middle Bronze at Tell el-Ajjul. In Annual of the Department of Antiquities of Jordan 3, 41–55.
Yavne	62-75	Burial	Southern Coastal plain	66	18.116143	39.654947	50.92	20.2	complete	2a	non	flat	Brosh, M. 1971. Yavne. Hadashot Arkheologiyot 40: 13 (Hebrew).

Yavne	62-87	Burial	Southern Coastal plain	65	10.250294	64.325922	94.51	24.5	complete	2a	non	flat	Brosh, M. 1971. Yavne. Hadashot Arkheologiyot 40: 13 (Hebrew).
Yehud	L1022-B10091	Burial	Southern Coastal plain	55	12.340075	27.591264	48.3	24.3	complete	1a	non	flat	Govrin, Y. 2015. NGSBA Archaeology III - Excavations at Yehud The 2008-2009 Seasons D. Ilan (ed). HEBREW UNION COLLEGE.
Yehud	L142-B1102	Burial	Southern Coastal plain	58	12.214235	39.920073	66.09	10.42	complete	1b	non	flat	Govrin, Y. 2015. NGSBA Archaeology III - Excavations at Yehud The 2008-2009 Seasons D. Ilan (ed). HEBREW UNION COLLEGE.
Yehud	L21-R66-6	Burial	Southern Coastal plain	62	12.265896	33.834933	52.65	25.44	complete	2a	nub	flat	Govrin, Y. 2015. NGSBA Archaeology III - Excavations at Yehud The 2008-2009 Seasons D. Ilan (ed). HEBREW UNION COLLEGE.
Yehud	L21-R83-6	Burial	Southern Coastal plain	62	18.097199	31.156362	78.75	15.26	complete	1a	non	flat	Govrin, Y. 2015. NGSBA Archaeology III - Excavations at Yehud The 2008-2009 Seasons D. Ilan (ed). HEBREW UNION COLLEGE.
Yehud	L415-B11026	Burial	Southern Coastal plain	54	11.5524	30.999451	81.05	11.95	complete	1b	nub	flat	Govrin, Y. 2015. NGSBA Archaeology III - Excavations at Yehud The 2008-2009 Seasons D. Ilan (ed). HEBREW UNION COLLEGE.
Yehud	L750-B7131	Burial	Southern Coastal plain	66	13.662676	38.756009	54.53	27.16	complete	2a	non	flat	Govrin, Y. 2015. NGSBA Archaeology III - Excavations at Yehud The 2008-2009 Seasons D. Ilan (ed). HEBREW UNION COLLEGE.
Yehud	L753-B7140	Burial	Southern Coastal plain	58	12.925	39.474172	92.97	30.3	complete	2a	non	flat	Govrin, Y. 2015. NGSBA Archaeology III - Excavations at Yehud The 2008-2009 Seasons D. Ilan (ed). HEBREW UNION COLLEGE.
Yehud	L804-B8009	Burial	Southern Coastal plain	56	14.747553	35.239622	49.59	17.79	complete	2a	non	flat	Govrin, Y. 2015. NGSBA Archaeology III - Excavations at Yehud The 2008-2009 Seasons D. Ilan (ed). HEBREW UNION COLLEGE.
Einan	83-1506	Burial	Upper Galilee	54	6.933668	23.129342	81.84	12.04	complete	1b	vertical	flat	Eisenberg, E. 1985. A Burial Cave of the Early Bronze Age IV (MB I) Near ʿEnan. In Atiqot 17, 59–74. Israel Antiquities Authority.
Einan	83-1507	Burial	Upper Galilee	62	9.441408	28.811698	80.02	14.51	complete	1b	vertical	flat	Eisenberg, E. 1985. A Burial Cave of the Early Bronze Age IV (MB I) Near ʿEnan. In Atiqot 17, 59–74. Israel Antiquities Authority.
Einan	83-1508	Burial	Upper Galilee	63	10.791265	41.267491	61.67	17.71	complete	1a	vertical	flat	Eisenberg, E. 1985. A Burial Cave of the Early Bronze Age IV (MB I) Near ʿEnan. In Atiqot 17, 59–74. Israel Antiquities Authority.
Hanita	73-784	Burial	Upper Galilee	59	7.944544	15.035104	55.85	16.8	complete	1b	vertical	flat	Yannai, E, & Rochman-Halperin, A. 2008. Burial caves from the Intermediate Bronze Age at Hanita. In ʿAtiqot 59, 1*-10*. (Hebrew): Israel Antiquities Athority.
Hanita	73-786	Burial	Upper Galilee	60	7.072258	44.186861	86.88	11.09	complete	1b	shelf	flat	Yannai, E, & Rochman-Halperin, A. 2008. Burial caves from the Intermediate Bronze Age at Hanita. In ʿAtiqot 59, 1*-10*. (Hebrew): Israel Antiquities Athority.
Hazor	27712-5	Settelment	Upper Galilee	64	9.328154	39.493083	57.69	28.43	complete	2a	shelf	flat	Bechar, S. 2017. The Intermediate Bronze Age Pottery. In A. Ben-Tor, S. Zuckerman, S. Bechar, & D. Sandhaus (eds) HAZOR VII - The 1990-2012 Excavations - The Bronze Age, 161–98. Jerusalem.
Hazor	49310	Settelment	Upper Galilee	54	10.484624	18.464102	51.43	21.54	complete	1a	non	round	Bechar, S. 2017. The Intermediate Bronze Age Pottery. In A. Ben-Tor, S. Zuckerman, S. Bechar, & D. Sandhaus (eds) HAZOR VII - The 1990-2012 Excavations - The Bronze Age, 161–98. Jerusalem.
Hazor	62200-2	Settelment	Upper Galilee	63	10.844277	52.672875	98.32	31.04	complete	2a	vertical	flat	Bechar, S. 2017. The Intermediate Bronze Age Pottery. In A. Ben-Tor, S. Zuckerman, S. Bechar, & D. Sandhaus (eds) HAZOR VII - The 1990-2012 Excavations - The Bronze Age, 161–98. Jerusalem.
Hazor	95134-3	Settelment	Upper Galilee	56	8.370586	24.704174	87.62	20.82	complete	1a	vertical	flat	Bechar, S. 2017. The Intermediate Bronze Age Pottery. In A. Ben-Tor, S. Zuckerman, S. Bechar, & D. Sandhaus (eds) HAZOR VII - The 1990-2012 Excavations - The Bronze Age, 161–98. Jerusalem.
Nahaf	95-2006	Burial	Upper Galilee	60	7.412391	25.11947	57.97	17.13	complete	1b	shelf	flat	Getzov, N. 1995. Tombs from the Early and Intermediate Bronze Age in the Western Galilee. *Atiqot* 27((Hebrew)): *1-*18.
Nahaf	95-2007	Burial	Upper Galilee	51	9.071751	21.068907	50.76	18.37	complete	1b	non	round	Getzov, N. 1995. Tombs from the Early and Intermediate Bronze Age in the Western Galilee. *Atiqot* 27((Hebrew)): *1-*18.
Qedesh	70-492	Burial	Upper Galilee	55	10.625337	50.324215	103.2	20.17	complete	1a	vertical	flat	Tadmor, M. 1978. A Cult Cave of the Middle Bronze Age I near Qedesh. Israel Exploration Journal 28(1–2): 1–30.
Qedesh	70-496	Burial	Upper Galilee	72	9.441957	38.080296	74.64	19.89	complete	1a	nub	flat	Tadmor, M. 1978. A Cult Cave of the Middle Bronze Age I near Qedesh. Israel Exploration Journal 28(1–2): 1–30.
Qedesh	70-497	Burial	Upper Galilee	63	9.827102	72.258704	94.71	19.16	complete	1a	shelf	flat	Tadmor, M. 1978. A Cult Cave of the Middle Bronze Age I near Qedesh. Israel Exploration Journal 28(1–2): 1–30.
Qedesh	70-498	Burial	Upper Galilee	64	6.125856	25.679049	53.57	18.43	complete	1a	vertical	flat	Tadmor, M. 1978. A Cult Cave of the Middle Bronze Age I near Qedesh. Israel Exploration Journal 28(1–2): 1–30.
Hazorea	92-726	Burial	Western Valleys	66	9.39525	33.820097	49.96	17.75	complete	1a	non	round	Meyerhof, EL. 1989. The Bronze age necropolis at Kibbutz Hazorea, Israel. BAR International Series.
Iron	3009-47-2	Burial	Western Valleys	71	10.862597	37.223036	50.28	26.01	complete	2a	non	round	Yannai, E. 2016. ʿEn Esur (ʿEin Asawir) II: Excavations at the Cemeteries. Jerusalem: Israel Antiquities Authority.
Iron	3009-47-3	Burial	Western Valleys	62	7.613096	40.960062	52.25	13.14	complete	1b	non	round	Yannai, E. 2016. ʿEn Esur (ʿEin Asawir) II: Excavations at the Cemeteries. Jerusalem: Israel Antiquities Authority.
Iron	3009-47-47018	Burial	Western Valleys	60	10.730269	34.723091	54.34	17.45	complete	1b	non	round	Yannai, E. 2016. ʿEn Esur (ʿEin Asawir) II: Excavations at the Cemeteries. Jerusalem: Israel Antiquities Authority.
Iron	95-2506	Burial	Western Valleys	63	15.851599	51.937695	63.55	16.15	complete	1a	non	round	Yannai, E. 2016. ʿEn Esur (ʿEin Asawir) II: Excavations at the Cemeteries. Jerusalem: Israel Antiquities Authority.
Iron	95-2507	Burial	Western Valleys	64	8.528761	28.320657	63.04	14.83	complete	1b	shelf	round	Yannai, E. 2016. ʿEn Esur (ʿEin Asawir) II: Excavations at the Cemeteries. Jerusalem: Israel Antiquities Authority.
Iron	95-2540	Burial	Western Valleys	67	11.545069	32.882215	56.77	20.47	complete	1a	non	round	Yannai, E. 2016. ʿEn Esur (ʿEin Asawir) II: Excavations at the Cemeteries. Jerusalem: Israel Antiquities Authority.
Iron	95-2542	Burial	Western Valleys	62	11.304492	33.938858	51.19	12.1	complete	1b	non	round	Yannai, E. 2016. ʿEn Esur (ʿEin Asawir) II: Excavations at the Cemeteries. Jerusalem: Israel Antiquities Authority.
Iron	95-2543	Burial	Western Valleys	58	7.818426	71.207731	89.09	12.17	complete	1b	non	flat	Yannai, E. 2016. ʿEn Esur (ʿEin Asawir) II: Excavations at the Cemeteries. Jerusalem: Israel Antiquities Authority.
Iron	95-2546	Burial	Western Valleys	58	8.748341	33.011036	55.28	11.37	complete	1b	non	round	Yannai, E. 2016. ʿEn Esur (ʿEin Asawir) II: Excavations at the Cemeteries. Jerusalem: Israel Antiquities Authority.
Iron	95-2548	Burial	Western Valleys	66	14.275759	38.95008	64.64	10.44	complete	1b	non	round	Yannai, E. 2016. ʿEn Esur (ʿEin Asawir) II: Excavations at the Cemeteries. Jerusalem: Israel Antiquities Authority.
Iron	95-2549	Burial	Western Valleys	54	6.77862	25.736404	56.13	19.27	complete	1a	shelf	round	Yannai, E. 2016. ʿEn Esur (ʿEin Asawir) II: Excavations at the Cemeteries. Jerusalem: Israel Antiquities Authority.
Iron	95-2550	Burial	Western Valleys	55	11.958042	24.505319	55.65	23.25	complete	1a	shelf	round	Yannai, E. 2016. ʿEn Esur (ʿEin Asawir) II: Excavations at the Cemeteries. Jerusalem: Israel Antiquities Authority.
Iron	95-2553	Burial	Western Valleys	60	8.900091	39.953435	56.73	14.67	complete	1b	non	round	Yannai, E. 2016. ʿEn Esur (ʿEin Asawir) II: Excavations at the Cemeteries. Jerusalem: Israel Antiquities Authority.
Megiddo	34_1565	Burial	Western Valleys	59	8.393893	25.76334	57.35	17.48	complete	1b	shelf	flat	Guy, PL. 1938. Megiddo Tombs. Chicago: Oriental Institute Publications.
Megiddo	34_2262	Burial	Western Valleys	54	6.419038	29.393243	53.95	11.25	complete	1b	shelf	round	Guy, PL. 1938. Megiddo Tombs. Chicago: Oriental Institute Publications.
Megiddo	34_2288	Burial	Western Valleys	46	9.131149	33.819334	47.89	9.98	complete	1b	shelf	round	Guy, PL. 1938. Megiddo Tombs. Chicago: Oriental Institute Publications.
Megiddo	34_2313	Burial	Western Valleys	64	7.593277	34.769408	85.84	16.17	complete	1b	shelf	flat	Guy, PL. 1938. Megiddo Tombs. Chicago: Oriental Institute Publications.
Megiddo	34_2314	Burial	Western Valleys	61	13.169938	45.537233	58.41	21.94	complete	1a	shelf	flat	Guy, PL. 1938. Megiddo Tombs. Chicago: Oriental Institute Publications.
Megiddo	39_544	Burial	Western Valleys	61	6.953302	34.07905	52.67	13.19	complete	1b	non	flat	Guy, PL. 1938. Megiddo Tombs. Chicago: Oriental Institute Publications.
Megiddo	I_2896	Burial	Western Valleys	55	7.613084	29.872487	29.46	13.62	complete	1b	shelf	round	Guy, PL. 1938. Megiddo Tombs. Chicago: Oriental Institute Publications.
Megiddo	I_2897	Burial	Western Valleys	64	9.728196	18.261609	59.37	13.4	complete	1b	shelf	round	Guy, PL. 1938. Megiddo Tombs. Chicago: Oriental Institute Publications.
Megiddo	I_2988	Burial	Western Valleys	57	8.321066	27.982692	79.87	17.61	complete	1a	shelf	flat	Guy, PL. 1938. Megiddo Tombs. Chicago: Oriental Institute Publications.
Megiddo	I_9546	Burial	Western Valleys	57	7.377458	67.598299	53.05	15.77	complete	1b	shelf	flat	Guy, PL. 1938. Megiddo Tombs. Chicago: Oriental Institute Publications.
