# Supplementary material for: Toward the identification of social signatures in ceramic production – An archaeological case study
Source: PLoS One. 2021 Jul 26;16(7):e0254766. doi: 10.1371/journal.pone.0254766 (PMC8312935; doi:10.1371/journal.pone.0254766)
Supplement: S2 Table — (PDF) [file pone.0254766.s002.pdf]

Jar's number (according to the study)	Context	Strartum/Level	Horizons	Landscape unit	Rim Radius	Base Radius	Rim Curve	Neck Curve	Exteranl volume	Completeness	Morphological Type	Handle type	Reference
Jar023	Arad	?	Horizon 2 (8th century)	Negev Valley	50	38.278319	10.124721	27.848298	49.7	complete	1b	4 handles	Aharoni, M. and Aharoni, Y. 1976. The Stratification of Judahite Sites in the 8th and 7th Centuries BCE. BASOR 224: 73–90.
Jar025	Arad	VI	Horizon 4 (early 6th)	Negev Valley	51		12.901517	38.750847		not complete	2a	2 handles	Aharoni, M. and Aharoni, Y. 1976. The Stratification of Judahite Sites in the 8th and 7th Centuries BCE. BASOR 224: 73–90.
Jar045	Beer Sheba	II	Horizon 3 (late 8th and 7th)	Negev Valley	57	31.63113175	12.872748	35.453606	60.89	complete	1a	4 handles	Data currently unavailable, publication process in progress
Jar116	Beth Shemesh	3	Horizon 2 (8th century)	Sorek Valley	59	24.16799418	16.971967	39.367521	50.7	complete	1b	4 handles	Bunimovitz, S., & Lederman, T. 2016. Tel Beth-Shemesh: A Border Community in Judah: Renewed Excavations 1990–2000: The Iron Age (Vol. 34). Penn State Press.
Jar117	Beth Shemesh	3	Horizon 2 (8th century)	Sorek Valley	49	56.39137243	15.852367	34.885311	49.25	complete	1b	4 handles	Bunimovitz, S., & Lederman, T. 2016. Tel Beth-Shemesh: A Border Community in Judah: Renewed Excavations 1990–2000: The Iron Age (Vol. 34). Penn State Press.
Jar118	Beth Shemesh	3	Horizon 2 (8th century)	Sorek Valley	58	47.05138706	14.599989	46.955013	49.77	complete	1b	4 handles	Bunimovitz, S., & Lederman, T. 2016. Tel Beth-Shemesh: A Border Community in Judah: Renewed Excavations 1990–2000: The Iron Age (Vol. 34). Penn State Press.
Jar119	Beth Shemesh	3	Horizon 2 (8th century)	Sorek Valley	54	27.59099692	12.939887	46.089406	50.03	complete	1b	4 handles	Bunimovitz, S., & Lederman, T. 2016. Tel Beth-Shemesh: A Border Community in Judah: Renewed Excavations 1990–2000: The Iron Age (Vol. 34). Penn State Press.
Jar120	Beth Shemesh	3	Horizon 2 (8th century)	Sorek Valley	49	64.5715973	12.347422	43.269117	56.93	complete	1a	4 handles	Bunimovitz, S., & Lederman, T. 2016. Tel Beth-Shemesh: A Border Community in Judah: Renewed Excavations 1990–2000: The Iron Age (Vol. 34). Penn State Press.
Jar121	Beth Shemesh	3	Horizon 2 (8th century)	Sorek Valley	47	66.52127232	11.438477	42.762481	60.55	complete	1a	4 handles	Bunimovitz, S., & Lederman, T. 2016. Tel Beth-Shemesh: A Border Community in Judah: Renewed Excavations 1990–2000: The Iron Age (Vol. 34). Penn State Press.
Jar122	Beth Shemesh	3	Horizon 2 (8th century)	Sorek Valley	40		11.392188	40.791666		not complete	1a	4 handles	Bunimovitz, S., & Lederman, T. 2016. Tel Beth-Shemesh: A Border Community in Judah: Renewed Excavations 1990–2000: The Iron Age (Vol. 34). Penn State Press.
Jar123	Beth Shemesh	3	Horizon 2 (8th century)	Sorek Valley	51		11.132344	41.439651		not complete	1b	4 handles	Bunimovitz, S., & Lederman, T. 2016. Tel Beth-Shemesh: A Border Community in Judah: Renewed Excavations 1990–2000: The Iron Age (Vol. 34). Penn State Press.
Jar100	City of David	10	Horizon 4 (early 6th)	Jerusalem	44	25.49390067	9.668146	41.205304	45.56	complete	1b	4 handles	Data currently unavailable, publication process in progress
Jar101	City of David	10	Horizon 4 (early 6th)	Jerusalem	50	44.84235227	12.216946	34.998484	51.46	complete	2b	4 handles	Data currently unavailable, publication process in progress
Jar102	City of David	10	Horizon 4 (early 6th)	Jerusalem	49	27.62773036	8.695575	39.663365	43.66	complete	2a	4 handles	Data currently unavailable, publication process in progress
Jar103	City of David	10	Horizon 4 (early 6th)	Jerusalem	48	48.09257113	11.019683	36.948813	43.75	complete	2a	4 handles	Data currently unavailable, publication process in progress
Jar104	City of David	10	Horizon 4 (early 6th)	Jerusalem	51	14.72781378	10.978464	36.984905	35.04	complete	2a	4 handles	Data currently unavailable, publication process in progress
Jar105	City of David	10	Horizon 4 (early 6th)	Jerusalem	50	50.3324423	8.94038	48.586403	43.06	complete	2a	4 handles	Data currently unavailable, publication process in progress
Jar110	City of David	10	Horizon 4 (early 6th)	Jerusalem	45	46.75922173	14.700824	42.652755	39.15	complete	2a	4 handles	Data currently unavailable, publication process in progress
Jar1	City of David	10	Horizon 4 (early 6th)	Jerusalem	53	16.36494575	8.370017	36.640764	43.74	complete	2a	4 handles	Data currently unavailable, publication process in progress
Jar2	City of David	10	Horizon 4 (early 6th)	Jerusalem	46	53.23546009	14.266801	40.134909	47.27	complete	2a	4 handles	Data currently unavailable, publication process in progress
Jar3	City of David	10	Horizon 4 (early 6th)	Jerusalem	47		12.366286	45.414149		not complete	2a	?	Data currently unavailable, publication process in progress
Jar4	City of David	10	Horizon 4 (early 6th)	Jerusalem	49	46.78362604	9.510734	40.499264	54.1	complete	2b	4 handles	Data currently unavailable, publication process in progress
Jar5	City of David	10	Horizon 4 (early 6th)	Jerusalem	54	25.46521119	11.686716	39.308192	50.17	complete	2b	4 handles	Data currently unavailable, publication process in progress
Jar6	City of David	10	Horizon 4 (early 6th)	Jerusalem	53	21.15526263	8.576327	46.345106	54.95	complete	2b	4 handles	Data currently unavailable, publication process in progress
Jar013	Ein Gedi	V	Horizon 4 (early 6th)	Judean Desert	51	52.45261822	12.229787	36.127997	54.45	complete	2b	4 handles	Data currently unavailable, publication process in progress
Jar020	Ein Gedi	V	Horizon 4 (early 6th)	Judean Desert	49		8.368782	41.147241		not complete	2a	4 handles	Data currently unavailable, publication process in progress
Jar021	Ein Gedi	V	Horizon 4 (early 6th)	Judean Desert	52		7.737214	39.773517		not complete	2b	4 handles	Data currently unavailable, publication process in progress
Jar202	Ein Gedi	V	Horizon 4 (early 6th)	Judean Desert	50	51.27510792	8.371863	48.07032	45.96	complete	2a	4 handles	Data currently unavailable, publication process in progress
Jar300	Gezer	VIA	Horizon 2 (8th century)	Ayalon Valley	47		22.954807	48.308193		not complete	1a	?	Gitin, S. 1990. Gezer III: A Ceramic Typology of the Late Iron II, Persian and Hellenistic Periods at Tell Gezer. Jerusalem.
Jar301	Gezer	VIA	Horizon 2 (8th century)	Ayalon Valley	48		20.479696	31.957464		not complete	1a	?	Gitin, S. 1990. Gezer III: A Ceramic Typology of the Late Iron II, Persian and Hellenistic Periods at Tell Gezer. Jerusalem.
Jar302	Gezer	VIA	Horizon 2 (8th century)	Ayalon Valley	53		14.525792	38.746321		not complete	1a	?	Gitin, S. 1990. Gezer III: A Ceramic Typology of the Late Iron II, Persian and Hellenistic Periods at Tell Gezer. Jerusalem.
Jar303	Gezer	VIA	Horizon 2 (8th century)	Ayalon Valley	52		13.073458	32.350391		not complete	1b	4 handles	Gitin, S. 1990. Gezer III: A Ceramic Typology of the Late Iron II, Persian and Hellenistic Periods at Tell Gezer. Jerusalem.
Jar304	Gezer	VIA	Horizon 2 (8th century)	Ayalon Valley	49		11.633716	42.67359		not complete	1a	?	Gitin, S. 1990. Gezer III: A Ceramic Typology of the Late Iron II, Persian and Hellenistic Periods at Tell Gezer. Jerusalem.
Jar305	Gezer	VIA	Horizon 2 (8th century)	Ayalon Valley	48	50.56756745	11.087593	39.653902	45.14	complete	1b	4 handles	Gitin, S. 1990. Gezer III: A Ceramic Typology of the Late Iron II, Persian and Hellenistic Periods at Tell Gezer. Jerusalem.
Jar306	Gezer	VIA	Horizon 2 (8th century)	Ayalon Valley	52	27.35170339	11.048042	52.454191	48.36	complete	1b	4 handles	Gitin, S. 1990. Gezer III: A Ceramic Typology of the Late Iron II, Persian and Hellenistic Periods at Tell Gezer. Jerusalem.
Jar307	Gezer	VIA	Horizon 2 (8th century)	Ayalon Valley	46	52.67074964	10.940347	40.958562	45.51	complete	1b	4 handles	Gitin, S. 1990. Gezer III: A Ceramic Typology of the Late Iron II, Persian and Hellenistic Periods at Tell Gezer. Jerusalem.
Jar308	Gezer	VIA	Horizon 2 (8th century)	Ayalon Valley	54	31.3442134	10.38158	49.734693	63.74	complete	1a	4 handles	Gitin, S. 1990. Gezer III: A Ceramic Typology of the Late Iron II, Persian and Hellenistic Periods at Tell Gezer. Jerusalem.
Jar309	Gezer	VIA	Horizon 2 (8th century)	Ayalon Valley	50	16.25399575	10.097625	48.120382	54.43	complete	1a	4 handles	Gitin, S. 1990. Gezer III: A Ceramic Typology of the Late Iron II, Persian and Hellenistic Periods at Tell Gezer. Jerusalem.
Jar310	Gezer	VIA	Horizon 2 (8th century)	Ayalon Valley	51		8.052293	43.131469		not complete	1a	4 handles	Gitin, S. 1990. Gezer III: A Ceramic Typology of the Late Iron II, Persian and Hellenistic Periods at Tell Gezer. Jerusalem.
Jar002	Ira	VII	Horizon 3 (late 8th and 7th)	Negev Valley	46	10.54488171	17.040575	38.098968	45.41	complete	1b	4 handles	Freud, L. 1999. Pottery: Iron Age. In: Beit-Arieh, I., ed. *Tel >Ira: A Stronghold in the Biblical Negev* (Monograph Series of the Institute of Archaeology of Tel Aviv University 15). Tel Aviv: 189–289.
Jar003	Ira	VI	Horizon 4 (early 6th)	Negev Valley	52		9.690869	29.154124		not complete	2a	4 handles	Freud, L. 1999. Pottery: Iron Age. In: Beit-Arieh, I., ed. *Tel >Ira: A Stronghold in the Biblical Negev* (Monograph Series of the Institute of Archaeology of Tel Aviv University 15). Tel Aviv: 189–289.
Jar004	Ira	VII	Horizon 3 (late 8th and 7th)	Negev Valley	47		11.383932	38.1857		not complete	1b	4 handles	Freud, L. 1999. Pottery: Iron Age. In: Beit-Arieh, I., ed. *Tel >Ira: A Stronghold in the Biblical Negev* (Monograph Series of the Institute of Archaeology of Tel Aviv University 15). Tel Aviv: 189–289.
Jar005	Ira	VII	Horizon 3 (late 8th and 7th)	Negev Valley	52	68.07454073	13.150224	32.180781	34.96	complete	1b	4 handles	Freud, L. 1999. Pottery: Iron Age. In: Beit-Arieh, I., ed. *Tel >Ira: A Stronghold in the Biblical Negev* (Monograph Series of the Institute of Archaeology of Tel Aviv University 15). Tel Aviv: 189–289.
Jar010	Ira	VII	Horizon 3 (late 8th and 7th)	Negev Valley	49	54.95260784	12.726513	31.14758	59.11	complete	1a	4 handles	Freud, L. 1999. Pottery: Iron Age. In: Beit-Arieh, I., ed. *Tel >Ira: A Stronghold in the Biblical Negev* (Monograph Series of the Institute of Archaeology of Tel Aviv University 15). Tel Aviv: 189–289.
Jar014	Ira	VI	Horizon 4 (early 6th)	Negev Valley	48		10.350757	34.295597		not complete	2b	4 handles	Freud, L. 1999. Pottery: Iron Age. In: Beit-Arieh, I., ed. *Tel >Ira: A Stronghold in the Biblical Negev* (Monograph Series of the Institute of Archaeology of Tel Aviv University 15). Tel Aviv: 189–289.
Jar058	Lachish	III	Horizon 3 (late 8th and 7th)	Lowland	52	35.03482029	23.133975	39.428947	48.44	complete	1b	4 handles	Zimhoni, O. 2004. The Pottery of Level III and II. In: Ussishkin, D. *The Renewed Archaeological Excavations at Lachish (1973–1994)* (Monograph Series of the Institute of Archaeology of Tel Aviv University 22). Tel Aviv: 1789–1899.

Jar059	Lachish	III	Horizon 3 (late 8th and 7th)	Lowland	52		17.681856	26.801724		not complete	1b	4 handles	Zimhoni, O. 2004. The Pottery of Level III and II. In: Ussishkin, D. *The Renewed Archaeological Excavations at Lachish (1973–1994)* (Monograph Series of the Institute of Archaeology of Tel Aviv University 22). Tel Aviv: 1789–1899.
Jar060	Lachish	III	Horizon 3 (late 8th and 7th)	Lowland	50	19.9432766	17.006341	52.544519	53.52	complete	1a	4 handles	Zimhoni, O. 2004. The Pottery of Level III and II. In: Ussishkin, D. *The Renewed Archaeological Excavations at Lachish (1973–1994)* (Monograph Series of the Institute of Archaeology of Tel Aviv University 22). Tel Aviv: 1789–1899.
Jar061	Lachish	III	Horizon 3 (late 8th and 7th)	Lowland	48	68.94839717	16.951966	33.152308	50.82	complete	1a	4 handles	Zimhoni, O. 2004. The Pottery of Level III and II. In: Ussishkin, D. *The Renewed Archaeological Excavations at Lachish (1973–1994)* (Monograph Series of the Institute of Archaeology of Tel Aviv University 22). Tel Aviv: 1789–1899.
Jar062	Lachish	III	Horizon 3 (late 8th and 7th)	Lowland	52	27.93864978	16.565895	53.250032	57.18	complete	1a	4 handles	Zimhoni, O. 2004. The Pottery of Level III and II. In: Ussishkin, D. *The Renewed Archaeological Excavations at Lachish (1973–1994)* (Monograph Series of the Institute of Archaeology of Tel Aviv University 22). Tel Aviv: 1789–1899.
Jar063	Lachish	III	Horizon 3 (late 8th and 7th)	Lowland	48	44.82078077	16.092007	42.266829	50.22	complete	1b	4 handles	Zimhoni, O. 2004. The Pottery of Level III and II. In: Ussishkin, D. *The Renewed Archaeological Excavations at Lachish (1973–1994)* (Monograph Series of the Institute of Archaeology of Tel Aviv University 22). Tel Aviv: 1789–1899.
Jar064	Lachish	III	Horizon 3 (late 8th and 7th)	Lowland	52	63.54828209	14.704412	50.860982	66.48	complete	1a	4 handles	Zimhoni, O. 2004. The Pottery of Level III and II. In: Ussishkin, D. *The Renewed Archaeological Excavations at Lachish (1973–1994)* (Monograph Series of the Institute of Archaeology of Tel Aviv University 22). Tel Aviv: 1789–1899.
Jar065	Lachish	III	Horizon 3 (late 8th and 7th)	Lowland	49	23.93105646	14.624029	36.596354	53.3	complete	1b	4 handles	Zimhoni, O. 2004. The Pottery of Level III and II. In: Ussishkin, D. *The Renewed Archaeological Excavations at Lachish (1973–1994)* (Monograph Series of the Institute of Archaeology of Tel Aviv University 22). Tel Aviv: 1789–1899.
Jar066	Lachish	III	Horizon 3 (late 8th and 7th)	Lowland	49		14.028202	33.094379		not complete	1a	4 handles	Zimhoni, O. 2004. The Pottery of Level III and II. In: Ussishkin, D. *The Renewed Archaeological Excavations at Lachish (1973–1994)* (Monograph Series of the Institute of Archaeology of Tel Aviv University 22). Tel Aviv: 1789–1899.
Jar069	Lachish	III	Horizon 3 (late 8th and 7th)	Lowland	51	32.20549493	13.977961	52.087301	49.77	complete	1b	4 handles	Zimhoni, O. 2004. The Pottery of Level III and II. In: Ussishkin, D. *The Renewed Archaeological Excavations at Lachish (1973–1994)* (Monograph Series of the Institute of Archaeology of Tel Aviv University 22). Tel Aviv: 1789–1899.
Jar071	Lachish	III	Horizon 3 (late 8th and 7th)	Lowland	49	16.48893822	13.788611	44.04379	62.32	complete	1a	4 handles	Zimhoni, O. 2004. The Pottery of Level III and II. In: Ussishkin, D. *The Renewed Archaeological Excavations at Lachish (1973–1994)* (Monograph Series of the Institute of Archaeology of Tel Aviv University 22). Tel Aviv: 1789–1899.
Jar072	Lachish	II	Horizon 4 (early 6th)	Lowland	53	18.94938268	13.494566	47.66094	46.76	complete	2a	4 handles	Zimhoni, O. 2004. The Pottery of Level III and II. In: Ussishkin, D. *The Renewed Archaeological Excavations at Lachish (1973–1994)* (Monograph Series of the Institute of Archaeology of Tel Aviv University 22). Tel Aviv: 1789–1899.
Jar073	Lachish	III	Horizon 3 (late 8th and 7th)	Lowland	47	53.96897953	13.326263	50.866542	55.1	complete	1a	4 handles	Zimhoni, O. 2004. The Pottery of Level III and II. In: Ussishkin, D. *The Renewed Archaeological Excavations at Lachish (1973–1994)* (Monograph Series of the Institute of Archaeology of Tel Aviv University 22). Tel Aviv: 1789–1899.
Jar074	Lachish	III	Horizon 3 (late 8th and 7th)	Lowland	56	66.32955013	13.304196	34.466392	57	complete	1a	4 handles	Zimhoni, O. 2004. The Pottery of Level III and II. In: Ussishkin, D. *The Renewed Archaeological Excavations at Lachish (1973–1994)* (Monograph Series of the Institute of Archaeology of Tel Aviv University 22). Tel Aviv: 1789–1899.
Jar078	Lachish	III	Horizon 3 (late 8th and 7th)	Lowland	54	57.76658866	13.242314	34.329763	52.43	complete	1b	4 handles	Zimhoni, O. 2004. The Pottery of Level III and II. In: Ussishkin, D. *The Renewed Archaeological Excavations at Lachish (1973–1994)* (Monograph Series of the Institute of Archaeology of Tel Aviv University 22). Tel Aviv: 1789–1899.
Jar081	Lachish	III	Horizon 3 (late 8th and 7th)	Lowland	53	68.30558776	13.214646	55.450583	62.63	complete	1a	4 handles	Zimhoni, O. 2004. The Pottery of Level III and II. In: Ussishkin, D. *The Renewed Archaeological Excavations at Lachish (1973–1994)* (Monograph Series of the Institute of Archaeology of Tel Aviv University 22). Tel Aviv: 1789–1899.
Jar082	Lachish	III	Horizon 3 (late 8th and 7th)	Lowland	51		12.795678	37.665685		not complete	1a	4 handles	Zimhoni, O. 2004. The Pottery of Level III and II. In: Ussishkin, D. *The Renewed Archaeological Excavations at Lachish (1973–1994)* (Monograph Series of the Institute of Archaeology of Tel Aviv University 22). Tel Aviv: 1789–1899.
Jar086	Lachish	III	Horizon 3 (late 8th and 7th)	Lowland	54	29.23074058	12.441875	35.037588	50.06	complete	1b	4 handles	Zimhoni, O. 2004. The Pottery of Level III and II. In: Ussishkin, D. *The Renewed Archaeological Excavations at Lachish (1973–1994)* (Monograph Series of the Institute of Archaeology of Tel Aviv University 22). Tel Aviv: 1789–1899.
Jar089	Lachish	III	Horizon 3 (late 8th and 7th)	Lowland	47	31.44683678	10.977425	27.4907	61.4	complete	1a	4 handles	Zimhoni, O. 2004. The Pottery of Level III and II. In: Ussishkin, D. *The Renewed Archaeological Excavations at Lachish (1973–1994)* (Monograph Series of the Institute of Archaeology of Tel Aviv University 22). Tel Aviv: 1789–1899.
Jar090	Lachish	III	Horizon 3 (late 8th and 7th)	Lowland	53		11.979538	34.371716		not complete	1b	4 handles	Zimhoni, O. 2004. The Pottery of Level III and II. In: Ussishkin, D. *The Renewed Archaeological Excavations at Lachish (1973–1994)* (Monograph Series of the Institute of Archaeology of Tel Aviv University 22). Tel Aviv: 1789–1899.
Jar093	Lachish	III	Horizon 3 (late 8th and 7th)	Lowland	47		11.206532	41.137275		not complete	1a	4 handles	Zimhoni, O. 2004. The Pottery of Level III and II. In: Ussishkin, D. *The Renewed Archaeological Excavations at Lachish (1973–1994)* (Monograph Series of the Institute of Archaeology of Tel Aviv University 22). Tel Aviv: 1789–1899.
Jar094	Lachish	III	Horizon 3 (late 8th and 7th)	Lowland	49	61.7344923	11.267679	30.672213	51.39	complete	1b	4 handles	Zimhoni, O. 2004. The Pottery of Level III and II. In: Ussishkin, D. *The Renewed Archaeological Excavations at Lachish (1973–1994)* (Monograph Series of the Institute of Archaeology of Tel Aviv University 22). Tel Aviv: 1789–1899.
Jar097	Lachish	II	Horizon 4 (early 6th)	Lowland	46	50.09508547	10.949521	43.082039	43.27	complete	2b	4 handles	Zimhoni, O. 2004. The Pottery of Level III and II. In: Ussishkin, D. *The Renewed Archaeological Excavations at Lachish (1973–1994)* (Monograph Series of the Institute of Archaeology of Tel Aviv University 22). Tel Aviv: 1789–1899.
Jar111	Lachish	III	Horizon 3 (late 8th and 7th)	Lowland	48		9.703116	69.126491		not complete	1a	4 handles	Zimhoni, O. 2004. The Pottery of Level III and II. In: Ussishkin, D. *The Renewed Archaeological Excavations at Lachish (1973–1994)* (Monograph Series of the Institute of Archaeology of Tel Aviv University 22). Tel Aviv: 1789–1899.

Jar112	Lachish	II	Horizon 4 (early 6th)	Lowland	50	47.71348046	10.863214	39.624012	48.71	complete	2b	4 handles	Zimhoni, O. 2004. The Pottery of Level III and II. In: Ussishkin, D. *The Renewed Archaeological Excavations at Lachish (1973–1994)* (Monograph Series of the Institute of Archaeology of Tel Aviv University 22). Tel Aviv: 1789–1899.
Jar126	Lachish	III	Horizon 3 (late 8th and 7th)	Lowland	47	21.46493705	10.344133	39.897112	43.19	complete	1b	4 handles	Zimhoni, O. 2004. The Pottery of Level III and II. In: Ussishkin, D. *The Renewed Archaeological Excavations at Lachish (1973–1994)* (Monograph Series of the Institute of Archaeology of Tel Aviv University 22). Tel Aviv: 1789–1899.
Jar201	Lachish	III	Horizon 3 (late 8th and 7th)	Lowland	51	12.18077501	9.976312	41.671885	49.08	complete	1b	4 handles	Zimhoni, O. 2004. The Pottery of Level III and II. In: Ussishkin, D. *The Renewed Archaeological Excavations at Lachish (1973–1994)* (Monograph Series of the Institute of Archaeology of Tel Aviv University 22). Tel Aviv: 1789–1899.
Jar203	Lachish	III	Horizon 3 (late 8th and 7th)	Lowland	51	27.27776953	9.857922	39.479164	54.14	complete	1a	4 handles	Zimhoni, O. 2004. The Pottery of Level III and II. In: Ussishkin, D. *The Renewed Archaeological Excavations at Lachish (1973–1994)* (Monograph Series of the Institute of Archaeology of Tel Aviv University 22). Tel Aviv: 1789–1899.
Jar204	Lachish	III	Horizon 3 (late 8th and 7th)	Lowland	48	38.33684207	8.919257	39.586457	59.69	complete	1a	4 handles	Zimhoni, O. 2004. The Pottery of Level III and II. In: Ussishkin, D. *The Renewed Archaeological Excavations at Lachish (1973–1994)* (Monograph Series of the Institute of Archaeology of Tel Aviv University 22). Tel Aviv: 1789–1899.
Jar205	Lachish	III	Horizon 3 (late 8th and 7th)	Lowland	50		7.825501	43.258629		not complete	1a	4 handles	Zimhoni, O. 2004. The Pottery of Level III and II. In: Ussishkin, D. *The Renewed Archaeological Excavations at Lachish (1973–1994)* (Monograph Series of the Institute of Archaeology of Tel Aviv University 22). Tel Aviv: 1789–1899.
Jar043	Malhata	III	Horizon 4 (early 6th)	Negev Valley	50	49.84308471	9.308521	41.584588	56.36	complete	2b	4 handles	Beit-Arieh, I. and Freud, L. 2015. *Tel Malḥata. A Central City in the Biblical Negev*. Monograph series of the Institute of Archaeology of Tel Aviv University 32. Vols. I, II: Winona Lake.
Jar032	Tel Batash	III	Horizon 3 (late 8th and 7th)	Sorek Valley	50	55.74653112	15.374443	52.653122	45.5	complete	1b	2 handles	Mazar, A. and Panitz-Cohen, N. 2001. *Timnah (Tel Batash) II: The Finds from the First Millennium BCE* (Qedem 42). Jerusalem.
Jar034	Tel Batash	II	Horizon 4 (early 6th)	Sorek Valley	51	39.50293167	13.201496	38.900571	47.61	complete	1b	4 handles	Mazar, A. and Panitz-Cohen, N. 2001. *Timnah (Tel Batash) II: The Finds from the First Millennium BCE* (Qedem 42). Jerusalem.
Jar035	Tel Batash	III	Horizon 3 (late 8th and 7th)	Sorek Valley	66	24.37821687	11.680035	60.098603	58.91	complete	1a	4 handles	Mazar, A. and Panitz-Cohen, N. 2001. *Timnah (Tel Batash) II: The Finds from the First Millennium BCE* (Qedem 42). Jerusalem.
Jar129	Tel Safi/Gath	A3	Horizon 1 (late 9th)	Lowland	44	20.13500266	20.758374	42.68808	58.69	complete	1a	4 handles	Shai, I. and Maeir, A.M. 2012. The Late Iron Age IIA Pottery Assemblage from Stratum A3. In: *Tell es-Safi/Gath I: The 1996 – 2005 Seasons*. Part 1: Text. Edited by Aren M. Maeir. HARRASSOWITZ VERLAG- WIESBADEN.
Jar130	Tel Safi/Gath	A3	Horizon 1 (late 9th)	Lowland	53		15.194506	44.875222		not complete	1b	4 handles	Shai, I. and Maeir, A.M. 2012. The Late Iron Age IIA Pottery Assemblage from Stratum A3. In: *Tell es-Safi/Gath I: The 1996 – 2005 Seasons*. Part 1: Text. Edited by Aren M. Maeir. HARRASSOWITZ VERLAG- WIESBADEN.
Jar131	Tel Safi/Gath	A3	Horizon 1 (late 9th)	Lowland	50	35.90790275	13.928164	40.169611	44.74	complete	1b	4 handles	Shai, I. and Maeir, A.M. 2012. The Late Iron Age IIA Pottery Assemblage from Stratum A3. In: *Tell es-Safi/Gath I: The 1996 – 2005 Seasons*. Part 1: Text. Edited by Aren M. Maeir. HARRASSOWITZ VERLAG- WIESBADEN.
Jar133	Tel Safi/Gath	A3	Horizon 1 (late 9th)	Lowland	45		12.841539	47.409822		not complete	1a	?	Shai, I. and Maeir, A.M. 2012. The Late Iron Age IIA Pottery Assemblage from Stratum A3. In: *Tell es-Safi/Gath I: The 1996 – 2005 Seasons*. Part 1: Text. Edited by Aren M. Maeir. HARRASSOWITZ VERLAG- WIESBADEN.
Jar134	Tel Safi/Gath	A3	Horizon 1 (late 9th)	Lowland	52		10.802156	49.423958		not complete	1a	?	Shai, I. and Maeir, A.M. 2012. The Late Iron Age IIA Pottery Assemblage from Stratum A3. In: *Tell es-Safi/Gath I: The 1996 – 2005 Seasons*. Part 1: Text. Edited by Aren M. Maeir. HARRASSOWITZ VERLAG- WIESBADEN.
Jar140	Tel Safi/Gath	A3	Horizon 1 (late 9th)	Lowland	54	12.57301101	8.868555	51.064901	61.66	complete	1a	4 handles	Shai, I. and Maeir, A.M. 2012. The Late Iron Age IIA Pottery Assemblage from Stratum A3. In: *Tell es-Safi/Gath I: The 1996 – 2005 Seasons*. Part 1: Text. Edited by Aren M. Maeir. HARRASSOWITZ VERLAG- WIESBADEN.
